# Gas Sensing Properties of a Novel Indium Oxide Monolayer: A First-Principles Study

**DOI:** 10.1021/acsomega.5c09366

**Published:** 2025-12-11

**Authors:** Afreen Anamul Haque, Suraj G. Dhongade, Aniket Singha

**Affiliations:** Department of Electronics and Electrical Communication Engineering, 30133 Indian Institute of Technology Kharagpur, Kharagpur 721302, India

## Abstract

We present a comprehensive first-principles investigation into the gas sensing capabilities of a novel two-dimensional (2D) Indium Oxide (In_2_O_3_) monolayer, using density functional theory (DFT) calculations. Targeting both resistive-type and work-function-based detection mechanisms, we evaluate the monolayer’s interactions with ten hazardous species, namely NH_3_, NO, NO_2_, SO_2_, CS_2_, H_2_S, HCN, CCl_2_O, CH_2_O, and CO. To assess the sensor’s deployability in ambient environments, we also analyze its interaction with common atmospheric or background gas molecules, such as, O_2_, CO_2_, and H_2_O. We note that NO and H_2_S molecules, with adsorption energy (*E*_ads_) of −0.68 and −1.29 eV respectively, can be detected via both substantial conductivity modulation (>10^6^×) and work-function shifts (Δϕ = 38.27 and 21.70% respectively). NH_3_ and HCN molecules, with *E*_ads_ = −1.07 and −0.46 eV respectively, on the other hand are readily detected through significant work-function alteration only (Δϕ = 25.38 and 17.80% respectively). Biaxial mechanical strain further proves highly effective in broadening the sensing capability, with tensile strain adjusting the adsorption energy favorably in most cases and additionaly facilitating the detection of NO_2_, CS_2_, CCl_2_O, and CO molecules through either conductivity modulation or work-function shifts. Compressive strain, on the contrary, facilitates detection of the CH_2_O molecule via work-function modulation. These results establish 2D In_2_O_3_ as a highly promising and tunable platform for next-generation miniaturized gas sensors suited for environmental monitoring and safety-critical applications.

## Graphical abstract

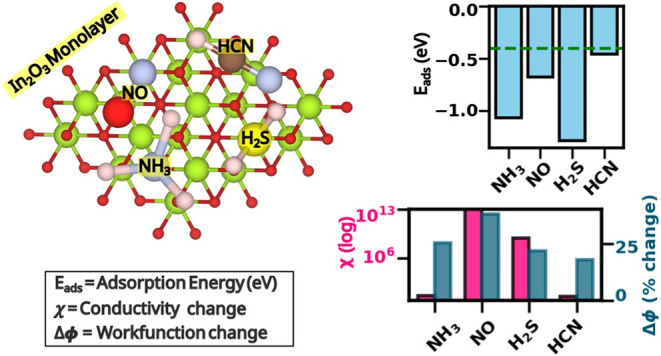

## 1 Introduction

Two-dimensional (2D) materials have emerged as a pivotal platform in contemporary nanotechnology, particularly since the isolation of monolayer graphene. Their growing prominence is attributed not only to their exceptional physical and chemical properties but also to their broad applicability across diverse technological domains. A defining feature of 2D systems is their ultrahigh surface-to-volume ratio, which significantly enhances their chemical reactivity and interaction with external species.^1–3^ Their electronic properties can also be precisely tuned via external stimuli, such as in-plane strain, electric fields, and chemical doping, offering considerable flexibility for device engineering. An additional advantage of 2D materials is their compositional tunability, which enables phase transitions among insulating, semiconducting, and metallic states through tailored elemental configurations.^4–6^ This versatility is further augmented by weak interlayer van der Waals forces, facilitating both surface-level and interlayer structural modifications. Such characteristics render 2D materials especially suitable for sensing applications, where attributes such as electronic responsiveness, chemical selectivity, and structural adaptability are of paramount importance.

Gas sensing, in particular, stands to benefit from the unique confluence of high electrical conductivity, mechanical flexibility, tunable electronic band structures, and functionalization potential inherent to 2D materials.^7,8^ Effective gas sensors are evaluated based on four key performance metrics: sensitivity, selectivity, response time, and operational stability. Sensitivity refers to the change in the measurable signal upon exposure to a target analyte relative to a baseline, while selectivity denotes the sensor’s ability to discriminate between the target gas and other ambient species.^9–12^ These macroscopic attributes are fundamentally governed by microscopic phenomena such as adsorption attributes, lattice distortions, and modifications in electronic properties such as the work function and density of states (DOS).

Motivated by recent theoretical predictions of stable layered In_2_O_3_ nanosheets,^13^ this work focuses on the gas sensing performance of a novel In_2_O_3_ monolayer, schematically depicted in Figure 1. Prior first-principles studies have established that this monolayer is a wide indirect bandgap semiconductor, with computed bandgaps of 1.64 eV (Perdew–Burke–Ernzerhof (PBE)) and 2.93 eV (HSE06).^13^ Interestingly, although intrinsically nonmagnetic, monolayer In_2_O_3_ has been predicted to exhibit ferromagnetism and half-metallicity upon hole doping, behavior arising from a Van Hove singularity near the valence band edge that leads to a Stoner instability. Monte Carlo simulations further estimate a Curie temperature of up to 62 K under moderate doping, underscoring the promise of In_2_O_3_ for future 2D spintronic and multifunctional sensing applications.

**Figure 1:**
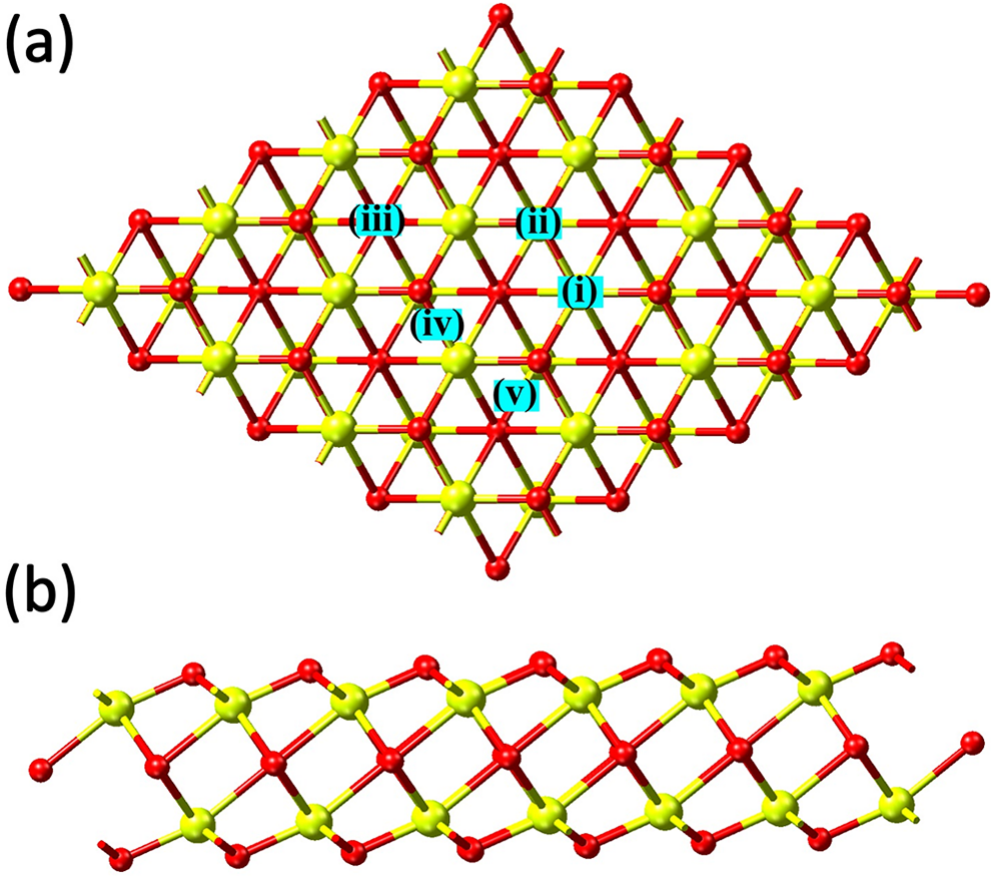
(a) Top and (b) side views of the novel pristine In_2_O_3_ monolayer. The high-symmetry sites considered for possible gas molecule adsorption are labeled with Roman numerals i–v in panel (a).

In this study, we present a comprehensive first-principles investigation of the gas sensing capabilities of the novel In_2_O_3_ monolayer, with particular emphasis on its adsorption characteristics and detection potential toward ten hazardous inorganic gases—NH_3_, NO, NO_2_, SO_2_, CS_2,_ H_2_S, HCN, CCl_2_O, CH_2_O, and CO. To evaluate the monolayer’s performance under ambient conditions, interactions with common background gases such as O_2_, CO_2_, and H_2_O are also examined. Due to the well-established chemical inertness of N_2_, its adsorption behavior is not considered in detail. The analysis aims to assess the viability of the In_2_O_3_ monolayer for application in both resistive-type and work-function-based gas sensing platforms.

The remainder of the paper is organized as follows. Section 2 describes the computational framework and parameters employed for computational analysis. Section 3 presents the core findings on gas adsorption, with Sections 3.1–3.3 addressing adsorption properties, resistive sensing response, and work-function-based sensing, respectively. Section 3.4 explores how mechanical strain can be employed to enhance sensing capabilities. A summary of our findings is presented in the conclusion in Section 4.

## 2 Computational Methods

We used the plane-wave-based Quantum Espresso suite for the simulations. Projector augmented wave (PAW) method, employing the generalized gradient approximations (GGA) in the Perdew–Burke–Ernzerhof parametrization (PBE), was employed for density functional theory (DFT) calculations. We have used a wave function cutoff of 60 Ry. Grimme’s DFT-D3 approach was used to describe nonlocal van der Waals interactions between the molecule-monolayer system.^14,15^ A 3 × 3 × 1 supercell of In_2_O_3_ in the *xy* plane is employed for investigation on the gas adsorption properties. To avoid spurious interactions between periodic images and correctly model the gas adsorption properties, a vacuum pad of 25 Å was employed in the *z*-direction. For iterative solution of the Kohn–Sham equations, the energy convergence threshold was set to be 10^–6^ Ry, and the forces on all atoms were converged until 10^–4^ Ry·Bohr^–1^. A 10 × 10 × 1 Monkhorst-grid of *k*-points was used to sample the Brillouin zone for structural optimization of the unit cell. The *k*-mesh was adapted accordingly for the supercell. For self-consistent field (SCF) and density of states calculations, a 25 × 25 × 1 Monkhorst-grid of *k*-mesh were used for the supercell. The above parameters like vacuum pad, *k*-mesh, and different convergence thresholds have been chosen based on the rigorous testing of total energy convergence with variation of these parameters. For work-function calculations, appropriate dipole corrections was employed to compute the local electrostatic potential along the *z*-direction.

## 3 Results and Discussion

For effective electrical gas sensing, a target molecule must establish a stable adsorption configuration with the sensing material, which generally requires an adsorption energy less than −15 *k*_B_ *T*, measuring around −0.4 eV at room temperature.^16,17^ Furthermore, the interaction between the gas molecule and the substrate should induce a detectable variation in the electrical property of the material. In this section, we present a predictive evaluation of the sensing capabilities of (i) resistive-type and (ii) work-function-type gas sensors based on the recently proposed In_2_O_3_ monolayer.^13^ For effective detection of the target gas in resistive-type sensors, the adsorption of the gas molecules should lead to the formation of shallow donor or acceptor states that effectively modulate the charge carrier concentration, thereby altering the electrical conductivity of the system.^6,18^ In contrast, work-function-type sensors operate on the principle of a change in the surface work function upon gas adsorption, which can be measured using a suitable apparatus, as discussed in Section 3.3. The following analysis of resistive- and work-function-type gas sensors based on In_2_O_3_ will focus on adsorption energetics, density of states (DOS), and surface potential modulation to determine the viability and response characteristics of the material under various gas exposures.

### 3.1 Adsorption Characteristics

As previously discussed, in both resistive and work-function-based gas sensing mechanisms, the interaction between gas molecules and the sensing surface requires the formation of a stable adsorbate–adsorbent complex with the underlying material. The adsorption energy serves as a crucial descriptor for evaluating the thermodynamic stability of adsorbed gas species and also impacts the adsorption height. Weak van der Waals forces typically drive physisorption, which is characterized by relatively low adsorption energy values. In contrast, chemisorption involves the formation of stronger chemical bonds, leading to significantly higher adsorption energy magnitudes. In addition to van der Waals forces and chemical bonds, induction of dipole in the monolayer by polar gas molecules can also influence the adsorption energy. To establish a realistic criterion for stable yet reversible adsorption at room temperature, a lower bound of −0.4 eV was adopted for the adsorption energy. This value is motivated by average thermal-kinetic energy considerations. At a temperature *T* = 300 K, the average kinetic energy of a gas molecule is ≈0.04 eV ( $\frac{3}{2}\;k_{B}T$ ). Adsorption energy of an order magnitude (10×) larger than this kinetic energy (≈0.4 eV) is generally sufficient to ensure that the molecules remain attached for sufficiently long duration to register a stable average measurable signal. Thus, in accordance with established literature, this study adopts a threshold adsorption energy of −0.4 eV as a criterion to determine whether a gas molecule is thermodynamically stably adsorbed on the surface of the monolayer.^16,19,20^ Adsorption energy beyond this threshold generally indicates unstable adsorption and recovery time which is too feeble for electrical detection. On the other hand, an adsorption energy exceeding −1 eV generally indicate strong binding with the monolayer surface and hints toward the necessity of thermal or UV treatment for recovery of the sensor layer after molecule adsorption.^16,19,20^ Furthermore, an adsorption energy exceeding −1.5 eV will generally indicate nonreusable (single-use) gas sensor or gas scavengers (used for trapping gas molecules or purifying environment). Hence, for our discussions, we will assume a suitable adsorption energy range of −0.4 to −1 eV for reusable gas sensors. The adsorption energy (*E*_ads_) was computed using the following relation

1 $$E_{\mathrm{ads}}=E_{\mathrm{molecule}+\mathrm{PL}}-E_{\mathrm{molecule}}-E_{\mathrm{PL}}$$

where *E*_molecule+PL_ is the total energy of the system comprising of the gas molecule adsorbed on the parent layer (PL), *E*_molecule_ is the energy of the isolated gas molecule, and *E*_PL_ is the energy of the pristine monolayer. Beyond adsorption energy, two additional metrics—adsorption height and recovery time—provide further insights into the interaction dynamics. The adsorption height represents the vertical distance between the adsorbed molecule and parent layer in the most stable configuration. To evaluate the reusability of the gas sensors, the recovery time (τ) was estimated via the Arrhenius-type equation

2 $$\tau =\frac{1}{\nu_{0}\;\exp\left(-E_{\mathrm{ads}}/k_{B}T\right)}$$

where ν_0_ denotes the attempt frequency (assumed as 10^12^ Hz), *k*_B_ is the Boltzmann constant, and *T* is the operating temperature in Kelvin. For practical sensor applications, a recovery time on the order of tens to hundreds of milli-seconds is generally preferred.^19,21^ Two-dimensional materials offer multiple potential sites for molecular adsorption. However, the energetically most favorable configuration, i.e., the one exhibiting the maximum magnitude of the adsorption energy, is typically considered the most stable. In this study, the favorable adsorption site for each gas molecule was systematically explored by initially placing the molecule at multiple strategic orientations (horizontal, vertical, and oblique at 45° angle) on the five high-symmetry positions of the In_2_O_3_ monolayer, as demonstrated in Figure 1 (details given in Section A1 of the Supporting Information). For polar molecules such as ammonia (NH_3_) and water (H_2_O), the effect of molecular dipole orientation was explored by aligning different molecular ends toward the surface. The most stable configuration, corresponding to the minimum adsorption energy, was identified and adopted for subsequent analysis (details provided in Section A1 of Supporting Information). The top and side views of the monolayer with adsorbed molecules in the most stable (minimum energy) configuration are demonstrated in Section A2 of the Supporting Information. The optimized adsorption energy, adsorption height, and recovery time for each gas molecule on the In_2_O_3_ monolayer is summarized in Table 1 and demonstrated graphically in Figure 2.

**Figure 2:**
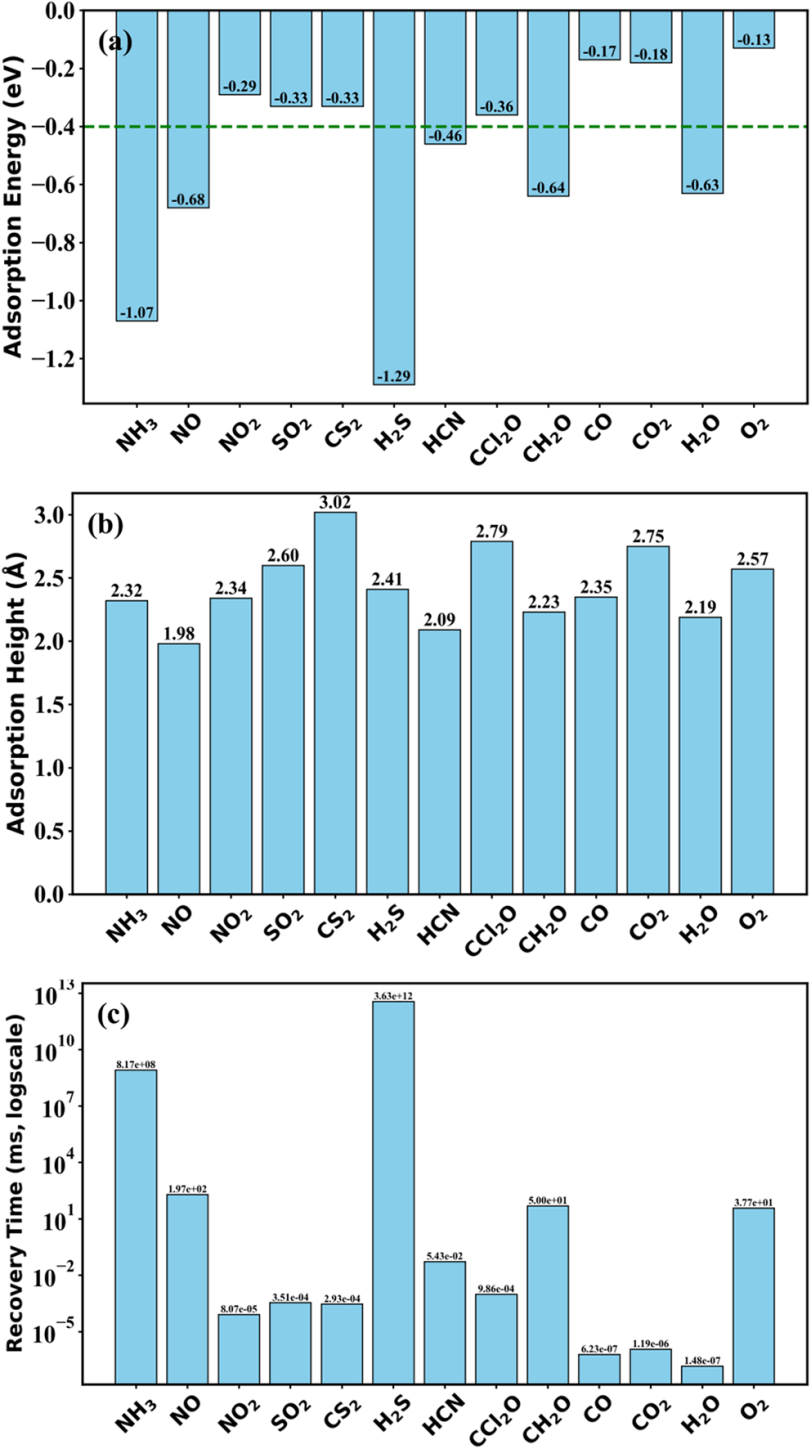
Adsorption characteristics for the considered molecules on the In_2_O_3_ monolayer. Bar graphs demonstrate (a) adsorption energy (eV), (b) adsorption height (Å), and (c) recovery time for all of the gas molecules under consideration.

**Table 1:** Values of Adsorption Energy (*E*_ads_), Adsorption Height (*H*), and Recovery Time (τ), for Different Molecules Investigated on Parent In_2_O_3_ Layer

molecule	*E*_ads_ (eV)	*H* (Å)	τ (ms)
NH_3_	–1.07	2.32	8.17 × 10^8^
NO	–0.68	1.98	197
NO_2_	–0.29	2.34	8.07 × 10^–5^
SO_2_	–0.33	2.60	3.51 × 10^–4^
CS_2_	–0.33	3.02	2.93 × 10^–4^
H_2_S	–1.29	2.41	3.63 × 10^12^
HCN	–0.46	2.09	5.43 × 10^–2^
CCl_2_O	–0.36	2.79	9.86 × 10^–4^
CH_2_O	–0.64	2.23	50.0
CO	–0.17	2.35	6.23 × 10^–7^
CO_2_	–0.18	2.75	1.19 × 10^–6^
H_2_O	–0.63	2.19	37.7
O_2_	–0.13	2.57	1.48 × 10^–7^

All 13 molecules examined in this study exhibit negative adsorption energy in their most energetically favorable configurations. This observation confirms the exothermic nature of the adsorption process. Based on the calculated adsorption energy given in Table 1, it is evident that NO_2_, SO_2_, CS_2_, CCl_2_O, CO, CO_2_, and O_2_ exhibit weak interactions with the monolayer, as their adsorption energy lie below the widely acknowledged threshold of −0.4 eV, suggesting that these molecules may not be effectively retained on the surface for reliable detection. In particular, CO, CO_2_, and O_2_ demonstrate adsorption energies of −0.17, −0.18, and −0.13 eV, along with adsorption heights of 2.35, 2.75, and 2.57 Å, respectively. NO_2_, SO_2_, CS_2_, and CCl_2_O exhibited slightly higher adsorption energy of −0.29, −0.33, −0.33, and −0.36 eV, respectively, still remaining under the threshold adsorption energy of −0.4 eV. The corresponding adsorption heights for NO_2_, SO_2_, CS_2_, and CCl_2_O are found to be 2.34, 2.60, 3.02, and 2.79 Å, respectively. It is important to note that recovery times for these molecules, of the order of one to tens of microseconds, are generally too brief to produce measurable changes in electrical conductivity or work function in commercial set-ups, thereby limiting the effectiveness of reliable gas detection in such cases.

HCN, with an adsorption energy of −0.46 eV and a height of 2.09 Å, demonstrates a relatively moderate interaction with the monolayer. Molecules such as CH_2_O and H_2_O exhibit adsorption energy of −0.64 and −0.63 eV, respectively, with moderate recovery times of 50 and 37.7 ms and adsorption heights around 2.2 Å, indicating that both gases are moderately bound yet can be readily desorbed, making them potential candidates for reusable room-temperature gas sensing with In_2_O_3_ monolayer. Among all molecules, NO shows the shortest adsorption height of 1.98 Å and a moderate adsorption energy of −0.68 eV, yet it induces minimal distortion in the monolayer structure. Its recovery time of 197 ms further supports its suitability for real-time sensing, striking an optimal balance between stability, and reusability.

The two molecules, NH_3_ and H_2_S, exhibit very high adsorption energies of −1.07 and −1.29 eV, respectively, and induce notable structural distortion, consistent with their higher adsorption energy. The observed dissociation of the H_2_S (details available in Figure S2 of Section A2 of the Supporting Information) molecule indicates that the H–S bond breaks during adsorption, resulting in covalent bond formation between the surface oxygen and the liberated hydrogen atom, while the remaining SH fragment interacts strongly with surface Indium atoms. The adsorption heights of these molecules are 2.32 and 2.41 Å respectively. Despite strong binding, their long recovery times of 8.17 × 10^8^ and 3.63 × 10^12^ ms pose significant limitations for practical reuse as reusable sensors without thermal or UV treatment.^19,22–24^ It is noteworthy that ambient molecule H_2_O exhibits a moderate adsorption energy of −0.63 eV, exceeding the threshold of −0.4 eV. This suggests a potential for competitive adsorption of H_2_O at the active sites, which may hinder the selective detection of target toxic gases. Therefore, the In_2_O_3_ monolayer sensor may not be suitable for deployment in humid environments.

For comparison, comprehensive tables of the adsorption energy and recovery times for the potential molecules, demonstrating adsorption energy beyond −0.4 eV (namely, NH_3_, NO, H_2_S, HCN, CH_2_O, and H_2_O) on various 2D materials (as reported in the literature), have been included in Section A3 of the Supporting Information.

### 3.2 Application in Resistive-Type Gas Sensing

A resistive-type gas sensor operates by detecting changes in electrical resistance caused by the adsorption of gas molecules onto its surface. This process of change in resistance may follow the formation of shallow donor or acceptor states in the bandgap, promoting additional electrons or holes into the conduction or valence bands, respectively, and thereby increasing the concentration of mobile charge carriers that modulate the system’s resistance. To gain insight into the sensing mechanism of the In_2_O_3_ monolayer, we analyze the corresponding changes in the density of states (DOS) upon gas adsorption. Figure 3 shows the total DOS (in gray), with the Fermi level denoted by a red dashed line. The resulting modifications in the DOS and relative shift in Fermi energy offer a qualitative understanding of the conductivity variations observed upon gas molecule interaction, as these directly influence carrier availability and transport properties.

**Figure 3:**
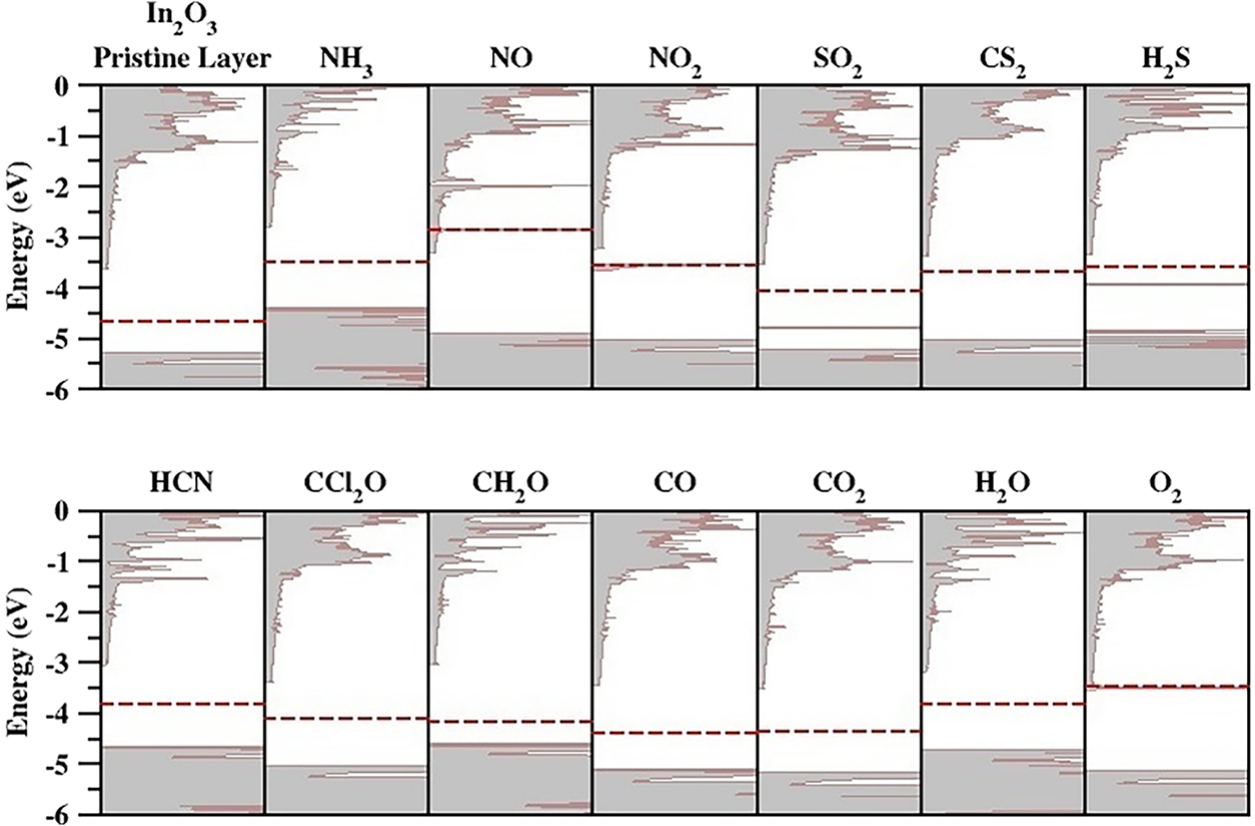
Total density of states (gray shaded region) for the pristine In_2_O_3_ monolayer and the combined molecule-monolayer systems. The Fermi level is shown by red dashed lines.

Figure 3 reveals that NO, and O_2_ adsorption induces metallic behavior in the 2D In_2_O_3_ monolayer. For NO and O_2_, the Fermi level penetrates the conduction band. These shifts suggest a substantial increase in electrical conductivity upon adsorption of the gas molecules. Among these, NO shows the most favorable sensing performance, with an adsorption energy of −0.68 eV making it highly effective for room-temperature detection. In the case of O_2_, the weak interaction, reflected by an adsorption energy of −0.13 eV and a rapid recovery time, limits its effectiveness for sensing under ambient conditions.

A pronounced change in conductivity is also expected upon NO_2_ adsorption, as the formation of a partially filled state in close proximity to the conduction band edge reduces the bandgap to 0.35 eV. This significant bandgap narrowing lowers the energy barrier for electron excitation into the conduction band, thereby promoting enhanced carrier generation and contributing to improved sensing response. However, the weak adsorption interaction, reflected by an adsorption energy of −0.29 eV and a fast recovery time, restricts its suitability for reliable sensing under ambient conditions. For H_2_S and SO_2_ adsorption, localized filled states emerge within the bandgap. Particularly, in the case of H_2_S, an induced state is located near the middle of the bandgap, slightly shifted toward the conduction band, whereas for SO_2_, the induced state appears close to the valence band maximum. The donor state generated by H_2_S, hence, effectively narrows the bandgap, introducing a moderately shallow defect level that facilitates electron excitation into the conduction band. However, the state induced by SO_2_ in the bandgap is a deep donor level and fails to significantly enhance conductivity at room temperature. H_2_S adsorption is also characterized by a high adsorption energy of −1.29 eV on the 2D In_2_O_3_ monolayer. Thus, the monolayer can detect H_2_S, but the high adsorption energy may hamper the reusability of the sensor unless subjected to thermal treatment or UV radiation for recovery. The adsorption of SO_2_ is relatively weak, with an adsorption energy of −0.33 eV, rendering its detection by the monolayer ineffective at room temperature.

In the cases of NH_3_, H_2_O, HCN, CO, CO_2_, CCl_2_O, CS_2_, and CH_2_O molecules, adsorption on the 2D In_2_O_3_ monolayer does not induce any shallow donor or acceptor states nor does it cause significant bandgap narrowing relative to the pristine system. As a result, the electronic structure remains largely unperturbed, and no substantial change in conductivity is expected upon adsorption of these molecules. Although NH_3_, CH_2_O and H_2_O exhibit a good adsorption energy of −1.07, −0.64, and −0.63 eV respectively, none of these molecules lead to the formation of electronically active states capable of enhancing charge carrier concentration at room temperature.

These results highlight the 2D In_2_O_3_ monolayer as a promising platform for resistive-type gas sensing, with pronounced sensitivity toward NO and H_2_S driven by adsorption-induced electronic modulation, while its limited response to other analytes ensures intrinsic selectivity.

#### 3.2.1 Conductivity Change Factor for the Adsorbed Systems

In several cases discussed above, the formation of induced empty or filled states within the bandgap of the pristine monolayer can facilitate enhanced conductivity. These states lower the required energy for electronic transitions from the highest occupied molecular orbital (HOMO) to the lowest unoccupied molecular orbital (LUMO), thereby enabling easier excitation of electrons into the conduction band or holes into the valence band. To quantitatively evaluate the electrical response of gas adsorption within the framework of resistive-type sensing, we define the conductivity change factor, χ, as the ratio of the electrical conductivity of the In_2_O_3_ monolayer with the adsorbed gas molecule to that of its pristine form. The electrical conductivity can be approximated using the expression

3 $$\sigma =AT^{3/2}e^{-E_{g}/2\;k_{B}T}$$

where *A* is a material-specific constant, *T* represents the absolute temperature, *k*_B_ is the Boltzmann constant, and *E*_g_ denotes the energy difference between the highest occupied molecular orbital (HOMO) and the lowest unoccupied molecular orbital (LUMO) for systems exhibiting semiconducting behavior.^21^

For molecule-monolayer systems exhibiting metallic character due to partially filled localized states within the bandgap, *E*_g_ is instead defined as the smaller of the two energy separations: between the induced state and the valence band maximum or between the induced state and the conduction band minimum. Since the conductivity of a material depends on the density of charge carriers (electrons in the conduction band and holes in the valence band), which are thermally activated across *E*_g_, any reduction in this gap upon gas adsorption enhances carrier excitation and thus increases conductivity. The corresponding conductivity change factor χ can be expressed as

4 $$\chi =\exp\left[\frac{-\left(E_{g}-E_{g}^{\prime }\right)}{2\;k_{B}T}\right]$$

where *E*_g_^′^ is the bandgap of the pristine In_2_O_3_ monolayer, and *E*_g_ corresponds to the modified bandgap upon gas adsorption as previously defined. The computed *E*_g_ and χ values for all adsorbed systems are summarized in Table 2. The electronic structure of pristine In_2_O_3_ was recalculated within our GGA-PBE (HSE) setup, yielding bandgap of 1.65 eV (2.98 eV) which is in excellent agreement with previously reported value of 1.64 eV (2.93 eV),^13^ thereby ensuring that the electronic properties and subsequent adsorption-induced DOS and work-function analyses are referenced to a consistent baseline. Since the intrinsic conductivity of the In_2_O_3_ monolayer is inherently low, we suggest that a conductivity change factor (χ) exceeding 10^6^ is necessary to enable effective detection in cost-efficient commercial devices. The lower limit of χ is imposed to ensure that for practical applications, the gas sensor exhibits sufficient selectivity toward specific toxic gas molecules. Such selectivity facilitates the identification of distinct species, rather than providing a general indication of overall gas mixture toxicity. Furthermore, this criterion guarantees that the sensor response for target analytes is pronounced enough to dominate over background noise, which may cause large alteration in the baseline signal.

**Table 2:** Values of *E*_g_ and χ for Different Molecules Investigated on the Parent Layer In_2_O_3_

molecule	*E*_g_ (eV)	χ
pristine In_2_O_3_	1.65	1.0
NH_3_	1.59	3.19
NO	0	very high
NO_2_	0.3	2.13 × 10^11^
SO_2_	1.24	2.76 × 10^3^
CS_2_	1.66	0.82
H_2_S	0.59	7.86 × 10^8^
HCN	1.60	2.63
CCl_2_O	1.66	0.82
CH_2_O	1.57	4.69
CO	1.65	1.0
CO_2_	1.65	1.0
H_2_O	1.55	6.91
O_2_	0	very high

For NO and O_2_ adsorption, eq 4 becomes inapplicable as the Fermi level shifts into the conduction band, inducing metallic behavior with intrinsically high conductivity. The NO_2_-adsorbed system demonstrates an exceptional conductivity modulation, with a change factor (χ) of 2.13 × 10^11^. In addition, for H_2_O adsorbed system χ takes the value of 7.86 × 10^8^, which when combined with its high adsorption energy translates into a strong detection potential on the monolayer. In contrast to the above molecules, SO_2_ induces a relatively modest conductivity change of ∼2.76 × 10^3^, which, given the inherently low conductivity of the pristine monolayer, may be insufficient for reliable detection in cost-effective commercial sensors. For the remaining analytes, the calculated χ values are very low (less than 10), rendering them unsuitable for resistive-type detection based on conductivity variations alone. As elaborated in Section 3.1, despite their strong conductivity response, the low adsorption energy of NO_2_ and O_2_ compromises their practical detectability via resistive-type sensing due to poor surface retention and rapid desorption. Consequently, among all of the studied gases, NO and H_2_S emerge as the most promising candidates for room-temperature resistive-type gas sensing, owing to their favorable combination of significant DOS modifications, large conductivity change factors, and adequate adsorption strengths. The preceding discussion highlight the results obtained from our GGA-PBE setup. The computed results and conclusion provided by hybrid HSE calculation, given in the Section A4 (Figure S4 and Table S7) of the Supporting Information, also demonstrate a similar trend as the GGA-PBE calculation.

### 3.3 Application in Work-Function-Type Gas Sensing

A critical parameter for evaluating the sensing capabilities of the 2D-In_2_O_3_ monolayer is the change in the work function (Φ) induced by gas adsorption. This metric reflects the shift in the surface electronic potential upon interaction with gas molecules and serves as an indicator of charge redistribution and dipole formation at the interface. The principle of this sensing mechanism is based on the Kelvin probe technique, where variations in the contact potential difference are used to measure the work function with high precision.

The work function is calculated as the difference between the vacuum potential and the Fermi level of the system, as shown in the equation

5 $$\phi =E_{\mathrm{vac}}-E_{f}$$

where ϕ is the work function, *E*_vac_ is the vacuum energy level, and *E*_f_ is the Fermi energy. For the pristine In_2_O_3_ monolayer, the computed work function is 4.84 eV. The change in work function between the pristine monolayer and the adsorbed system quantifies the sensor’s response, with larger shifts indicating higher sensitivity. In our work, a threshold of 15% work-function change on the adsorption of the gas molecule is considered the minimum detectable change. As in the previous case, such a threshold is chosen to ensure that the monolayer remains sufficiently selective to a specific set of gas molecules and to aid in filtering-out a change in signal due to background noise. In addition, very small work-function shifts may be typically insufficient for reliable detection with conventional low-cost set-ups.

Figure 4 and Table 3 demonstrate the absolute work function of the gas-adsorbed monolayer as well as the percentage change in work function with respect to the pristine layer. It can be noted that the adsorption of NO induces the highest work-function change of 38.27%, with a calculated value of 2.99 eV, emphasizing the high sensitivity of the 2D-In_2_O_3_ monolayer for NO detection. Additionally, adsorption of NH_3_, NO_2_, CS_2_, H_2_S, HCN, H_2_O, and O_2_ results in work-function changes exceeding 15%, with values of 25.38, 23.23, 20.24, 21.7%, 17.8, 19.77, and 24.02%, respectively. Out of these molecules, NH_3_, NO, H_2_S, HCN, and H_2_O have adsorption energy in excess of −0.4 eV, based on the adsorption energy profile reported in Section 3.1. Hence, out of the toxic gas molecules, NH_3_, NO, H_2_S, and HCN can be detected using the work-function-based sensing mechanism. Notably, NH_3_ and HCN, which remained undetectable using resistance-based methods, now exhibit measurable detection sensitivity through the work-function-based approach, owing to their high adsorption energy. In addition, since H_2_O possess moderate adsorption energy and demonstrates suitable work-function change (>15%), the monolayer should not be used as a toxic gas sensor in humid environment, unless the focus is toward detection of H_2_O. The average potential profiles of the pristine and gas-adsorbed In_2_O_3_ monolayer are demonstrated in Section A7 and Figure S7 of the Supporting Information.

**Figure 4:**
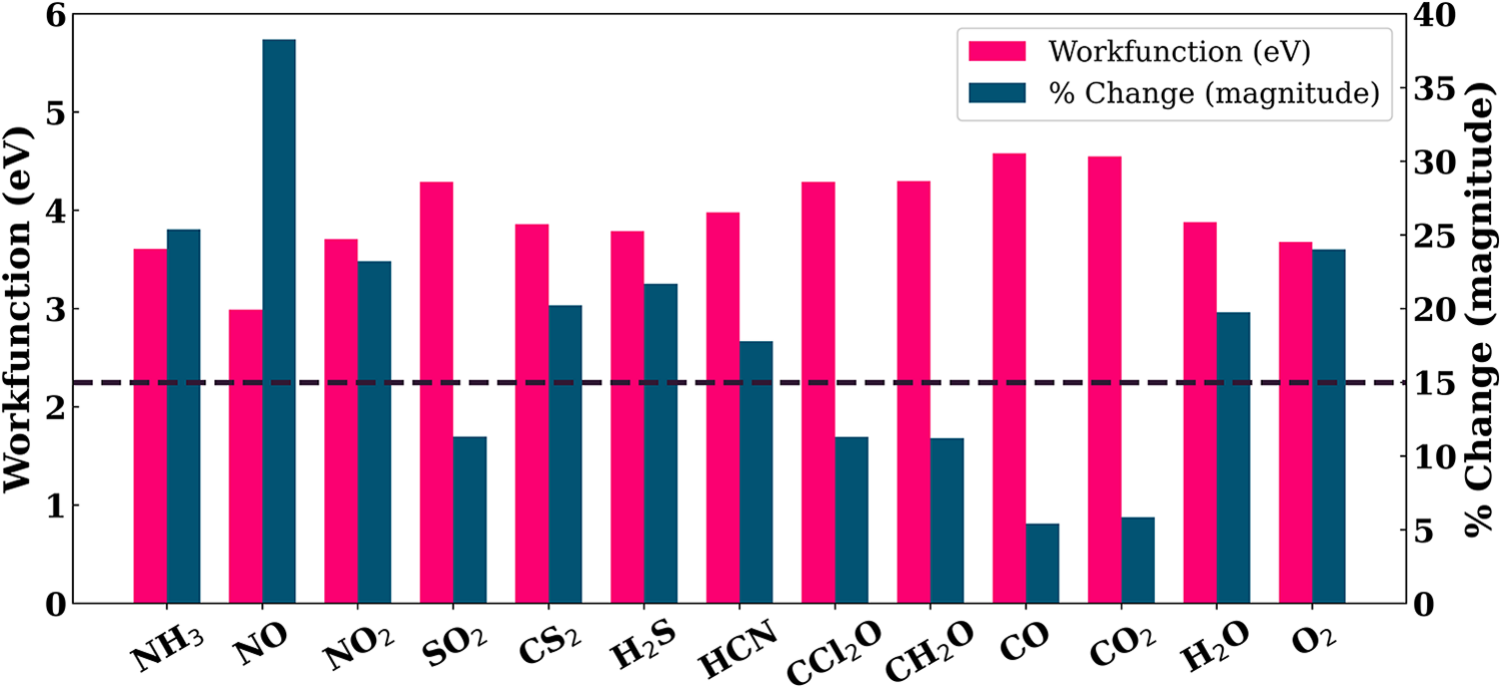
Percentage change in the work function of the In_2_O_3_ monolayer upon adsorption of gas molecules, compared to the pristine monolayer. The magenta dashed line represents the minimum percentage change in work function required for efficient detection.

**Table 3:** Computed Work-Function Values for All Gas-Adsorbed Configurations, along with Their Respective Percentage Deviations from the Pristine In_2_O_3_ Monolayer

molecule	work function (eV)	% change
pristine In_2_O_3_	4.84	0
NH_3_	3.61	25.38
NO	2.99	38.27
NO_2_	3.71	23.23
SO_2_	4.29	11.33
CS_2_	3.86	20.24
H_2_S	3.79	21.70
HCN	3.98	17.80
CCl_2_O	4.29	11.30
CH_2_O	4.30	11.21
CO	4.58	5.43
CO_2_	4.55	5.86
H_2_O	3.88	19.77
O_2_	3.68	24.02

### 3.4 Effect of Mechanical Strain on Sensing Performance

To explore the possibility of further enhancing the gas sensing performance in the In_2_O_3_ monolayer, we explore the effect of mechanical strain on the sensor performance. Strain-driven modulation of sensitivity is a widely recognized approach in 2D materials research, capable of substantially altering adsorption energetics and electronic structure. In this study, an in-plane biaxial tensile and compressive strain (up to ±5%) was applied to the monolayer to explore its effect on the sensing performance, particularly focusing on changes in adsorption energy and sensing characteristics. In what follows, we discuss the impact of strain on the adsorption energies of the gas molecules under consideration on the monolayer, and how strain influences their detectability in both resistive-type and work-function-based gas sensing mechanisms.

#### 3.4.1 Strain-Induced Modulation of the Adsorption Energy

It was observed that with a tensile strain of approximately 4%, the adsorption energies of O_2_ and CO_2_ were modulated to −0.47 and −0.54 eV, both exceeding the normally accepted threshold adsorption energy of −0.4 eV for suitable electrical detection. Given that O_2_ is an abundant ambient gas, and that oxygen-adsorbed In_2_O_3_ exhibits metallic behavior according to its density of states profile, such moderate adsorption may compromise the sensor’s selectivity by enabling unintended detection of background ambient gases. Moreover, with such moderate adsorption energy, the persistent presence of O_2_ at the adsorption sites could obstruct the binding of more relevant target analytes, thereby deteriorating the sensor’s efficiency and overall detection accuracy. Hence, we conclude that a moderate tensile strain of up to 3% is appropriate for investing the potential toward enhanced sensing of the harmful gases under consideration. Similarly, a compressive strain of 3% or more enhances the adsorption energy of H_2_O beyond −1 eV, which may result in H_2_O molecules to cling for excessively long duration to the active adsorption sites in the monolayer and hamper the detection of harmful gases. Thus, we conclude that a compressive strain of up to 2% can be suitably applied to the monolayer.

Figure 5 demonstrates the changes in adsorption energy for the 13 gas molecules under consideration with 3% tensile strain and 2% compressive strain applied to the monolayer. The exact values of the adsorption energy on the monolayer with 3% tensile strain and 2% compressive strain are documented in Table 4. It is evident that the application of 3% tensile strain significantly improves the adsorption behavior of several gas molecules on the In_2_O_3_ monolayer. In addition to molecules such as NH_3_, NO, H_2_S, HCN, CH_2_O, and H_2_O that already exhibited adsorption energies exceeding −0.4 eV in the unstrained monolayer, molecules such as NO_2_, SO_2_, CS_2_, CCl_2_O, and CO achieved adsorption energies exceeding −0.4 eV under 3% tensile strain, indicating stable adsorbate–adsorbent configuration for suitable detection. Among these molecules, SO_2_ stands out, with its adsorption energy increasing sharply to −1.03 eV. The other molecules NO_2_, CS_2_, CCl_2_O, and CO demonstrate adsorption energies of −0.5 eV, −0.51, −0.59, and −0.43 eV with 3% tensile strain. Interestingly, H_2_S, which initially exhibited an adsorption energy of −1.29 eV on the unstrained monolayer, now shows a reduced adsorption energy of −0.72 eV on the monolayer with 3% tensile strain, rendering it more suitable for a reusable gas sensor. In addition, the adsorption energy of NO increases from −0.68 to −0.96 eV under 3% tensile strain. We also note an undesirable modest enhancement in the adsorption energy for H_2_O molecule, from −0.63 to −0.73 eV, due to the applied +3% tensile strain. However, such strain-induced modulation of adsorption energy for H_2_O is significantly smaller than that observed for the target analytes (e.g., an improvement of −0.68 to −0.96 eV for NO; −0.33 to −1.03 eV for SO_2_; and −1.29 to −0.72 eV for H_2_S), thereby ensuring that moderate strain application favors the In_2_O_3_ monolayer’s role as a reusable and selective gas sensor. Furthermore, DOS analysis reveals that H_2_O adsorption does not introduce shallow donor or acceptor states within the bandgap, indicating negligible influence on electrical conductivity. Thus, while limited competition for the adsorption of active sites may occur from H_2_O molecules, it does not compromise the selective detection capability of the In_2_O_3_ monolayer, particularly under controlled or moderately humid conditions.

**Figure 5:**
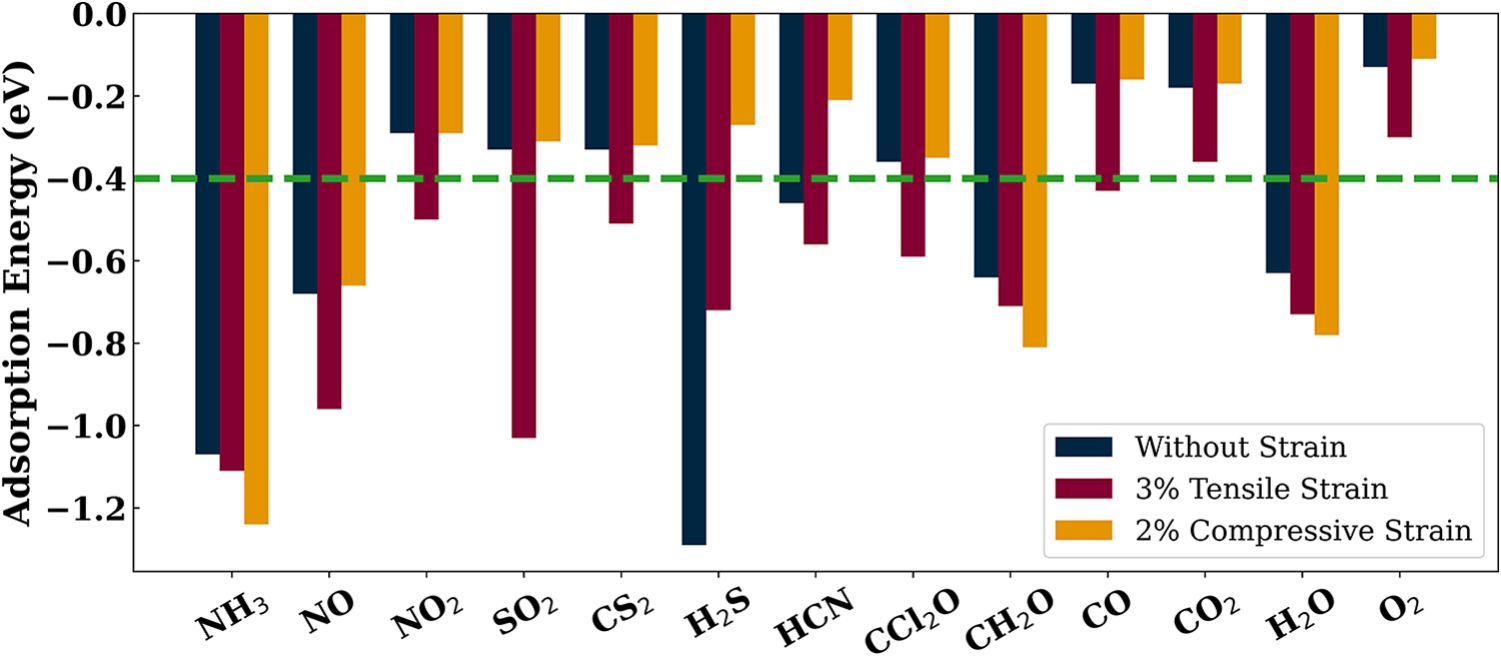
Adsorption energies of gas molecules on the In_2_O_3_ monolayer for three cases: unstrained (blue), with 3% tensile strain (maroon), and with 2% compressive strain (orange). The green dashed line represents the threshold value of 1 × 10 *k*_B_ *T* (eV).

**Table 4:** Adsorption Energies (eV) of Gas Molecules on the In_2_O_3_ Monolayer under Different Strain Conditions

	adsorption energy (eV)		
molecule	no strain	+3% strain	–2% strain
NH_3_	–1.07	–1.11	–1.24
NO	–0.68	–0.96	–0.66
NO_2_	–0.29	–0.50	–0.29
SO_2_	–0.33	–1.03	–0.31
CS_2_	–0.33	–0.51	–0.32
H_2_S	–1.29	–0.72	–0.27
HCN	–0.46	–0.56	–0.21
CCl_2_O	–0.36	–0.59	–0.35
CH_2_O	–0.64	–0.71	–0.81
CO	–0.17	–0.43	–0.16
CO_2_	–0.18	–0.36	–0.17
H_2_O	–0.63	–0.73	–0.78
O_2_	–0.13	0.30	–0.11

With a 2% compressive strain applied to the In_2_O_3_ monolayer, most gas molecules exhibit the same or a decrease in adsorption energy, with several falling below the threshold value of −0.4 eV, except NH_3_, CH_2_O, and H_2_O. These molecules, namely NH_3_, CH_2_O, and H_2_O, exhibit increased adsorption energies of −1.24, −0.81, and −0.78 eV, respectively.

#### 3.4.2 Resistive-Type Gas Sensing with Applied Strain

The density of states of the monolayer without and with the adsorbed gas molecules under 3% tensile strain and 2% compressive strain are demonstrated in Sections A5 and A6 (Figures S5 and S6) of the Supporting Material. Among the molecules, NO_2_, SO_2_, CS_2_, CCl_2_O, and CO that demonstrated crossing the threshold adsorption energy of −0.4 eV at 3% tensile strain, NO_2_ adsorption features an induced filled state near the edge of the conduction band. This induced state thus acts as a shallow donor and enhances the conductivity. The bandgap between the induced state and the conduction band edge was found to be 0.3 eV, giving rise to a large conductivity change factor (χ) of almost 2.13 × 10^11^. CS_2_ adsorption demonstrates a filled state in the bandgap of the pristine In_2_O_3_, which modifies the effective bandgap to 0.76 eV, increasing the conductivity of the monolayer by an approximate factor (χ) of 5 × 10^4^. However, since the monolayer has very low conductivity to begin with, such an enhancement in conductivity might not be suitable for detection with a commercially deployable low-cost setup. The other molecules, such as SO_2_, CCl_2_O, and CO adsorption, on the other hand, do not demonstrate suitable features in the DOS profile for electrical detection.

On the application of 2% compressive strain, no new molecule crosses the adsorption energy threshold of −0.4 eV. Hence, compressive strain does not offer any significant advantage for gas sensing applications with the In_2_O_3_ monolayer.

Thus, we conclude that the application of 3% tensile strain to the In_2_O_3_ monolayer aids the detection toward NO_2_ molecules, which remain undetectable in the unstrained configuration. Additionally, the tensile strain reduces the adsorption energy of the H_2_S molecule to a moderate range, thereby ensuring the sensor’s reusability for H_2_S detection. In contrast, the application of compressive strain does not confer any appreciable benefit to gas sensing applications. The average potential profiles of the pristine and gas-adsorbed strained In_2_O_3_ monolayer are demonstrated in Sections A8 and A9 (Figures S8 and S9) of the Supporting Information.

#### 3.4.3 Work-Function-Type Gas Sensing with Applied Strain

The absolute work-function values and their percentage changes, as compared to the strained monolayer before gas adsorption, for both tensile and compressive strains, are presented in Table 5 and graphically in Figure 6. Among the molecules, NO_2_, SO_2_, CS_2_, CCl_2_O, and CO, that exhibited adsorption energies exceeding the threshold of −0.4 eV under 3% tensile strain, NO_2_ adsorption is characterized by a work-function change of 19.36%. SO_2_ molecules result in a work-function of 4.65%, which is below the threshold value of 15% as considered in our paper. A 14.83% change in the work-function for CS_2_ adsorption is very close to the threshold work-function change of 15%. Thus, it can be considered as a detectable gas by work-function-type sensing arrangement. The remaining molecules, that is, CCl_2_O and CO, result in a work-function change of 22.94 and 18.21%, respectively, when adsorbed on the surface of the monolayer. Thus, the four molecules, namely, NO_2_, CS_2_, CCl_2_O, and CO, in addition to NH_3_, NO, H_2_S, and HCN molecules, can be detected by work-function-type sensing arrangement with the strained monolayer. Similar to the unstrained monolayer, H_2_O, with a work-function change of 16.49% and adsorption energy of −0.73 eV with 3% tensile strain, also shows potential for detection. Hence, the strained monolayer sensor should not be employed in humid environment unless the objective is toward moisture detection.

**Figure 6:**
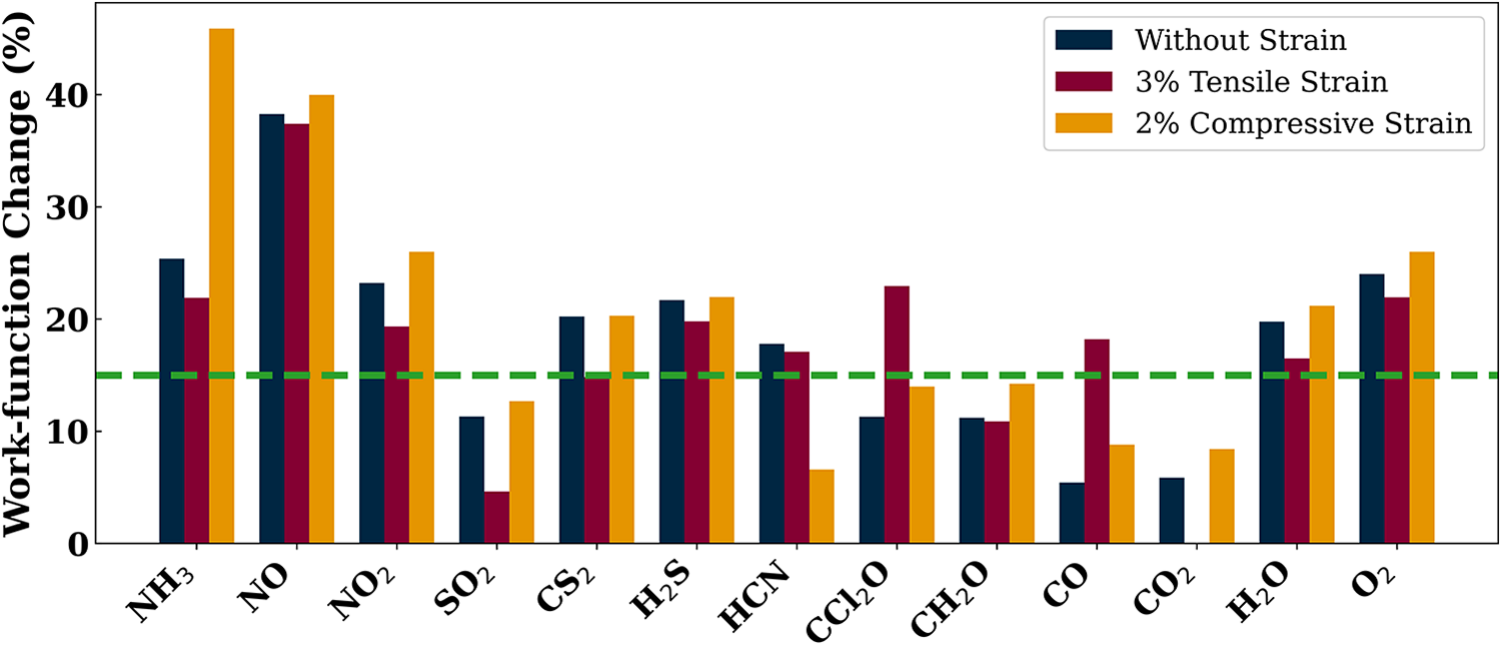
Percentage change in the work function of the In_2_O_3_ monolayer upon adsorption of gas molecules, compared to the pristine monolayer, for three cases: unstrained (blue), with 3% tensile strain (maroon), and with 2% compressive strain (orange). The green dashed line represents the minimum percentage change in work function required for efficient detection.

**Table 5:** Work-Function Values and Percentage Changes for Various Molecules under Tensile and Compressive Strain

	+3% strain		–2% strain	
molecule	W.F. (eV)	% change	W.F. (eV)	% change
In_2_O_3_	4.76		4.85	
NH_3_	3.71	21.90	2.62	45.90
NO	2.98	37.40	2.91	39.98
NO_2_	3.84	19.36	3.59	26.01
SO_2_	4.54	4.65	4.23	12.69
CS_2_	4.05	14.83	3.86	20.31
H_2_S	3.81	19.80	3.78	21.96
HCN	3.94	17.09	5.17	6.60
CCl_2_O	3.67	22.94	4.17	13.98
CH_2_O	4.24	10.88	4.16	14.24
CO	3.89	18.21	4.42	8.81
CO_2_	4.76	0.02	4.44	8.43
H_2_O	3.97	16.49	3.82	21.20
O_2_	3.71	21.94	3.59	26.01

As discussed earlier, the introduction of 2% compressive strain does not result in any additional molecules surpassing the adsorption energy threshold of −0.4 eV. However, for NH_3_, which demonstrated adsorption energy and work-function change of −1.07 eV and 25.38%, respectively, in the unstrained monolayer, now demonstrates adsorption energy of −1.24 eV and work-function change of 45.90%. Thus, under 2% compressive strain, the detectability of the NH_3_ molecule may improve, albeit at the expense of compromising the sensor’s reusability. Interestingly, CH_2_O exhibits an adsorption energy of −0.81 eV along with a work-function change of 14.24% (very close to the 15% threshold work-function change) when adsorbed on the monolayer under 2% compressive strain. It is noteworthy that CH_2_O, which remained undetectable by both resistive and work-function-based sensing mechanisms in the unstrained and tensile-strained monolayers, becomes detectable under compressive strain via a work-function-based sensing arrangement, provided a sufficiently sensitive measurement setup is employed.

## 4 Conclusions

In summary, this study comprehensively evaluated the In_2_O_3_ monolayer’s sensing capabilities by investigating its interactions with ten hazardous gases, namely NH_3_, NO, NO_2_, SO_2_, CS_2_, H_2_S, HCN, CCl_2_O, CH_2_O, and CO, while targeting both resistive-type and work-function-based detection pathways. To ensure practical deployability, the monolayer’s response to common atmospheric species, including O_2_, CO_2_, and H_2_O, was also thoroughly assessed, providing critical insights into its real-world deployment. The analysis reveals that NO and H_2_S adsorption exhibit strong adsorption energies, substantial modifications in the electronic density of states, and significant conductivity changes, positioning them as the most promising candidates for resistive-type sensing at room temperature. Work-function-based sensing extends the detection capability to additional analytes such as NH_3_ and HCN, which remain undetectable through resistive sensing alone.

Moving further, mechanical strain engineering was demonstrated as an effective strategy to enhance the sensing performance and selectivity. Under moderate tensile and compressive strains, several previously weakly adsorbing gases, like NO_2_, CS_2_, CCl_2_O, CH_2_O, and CO, exhibit improved adsorption energies and increased sensing responses either by resistance-based or by work-function-based sensing mechanism. Although the monolayer remains largely unresponsive to ambient gases like O_2_ and CO_2_, competitive H_2_O adsorption underscores the necessity for controlled humidity during practical sensor deployment. Overall, the 2D In_2_O_3_ monolayer offers a highly versatile platform for selective, sensitive, and potentially reusable gas sensing devices suitable for environmental monitoring and safety-critical applications.

## Supplementary Material



## Data Availability

The data supporting this article have been included as part of the main manuscript and Supporting Information.

## Notes

(i) Methodology for initial adsorption site selection and structural optimization (ii) Top and side views of adsorbed toxic gas molecules, (iii) Top and side views of adsorbed atmospheric gas molecules, (iv) Comparison of adsorption energies and recovery times from literature, (v) DOS profiles and conductivity change factor using hybrid HSE, (vi) DOS profiles under 3% tensile strain, (vii) DOS profiles under 2% compressive strain, (viii) Average potential profiles without strain, (ix) Average potential profiles under 3%, and (x) Average potential profiles under 2% compressive strain (PDF)
