# Supplementary material for: Gas Sensing Properties of a Novel Indium Oxide Monolayer: A First-Principles Study
Source: ACS Omega. 2025 Dec 11;10(50):62116–25. doi: 10.1021/acsomega.5c09366 (PMC12750183; doi:10.1021/acsomega.5c09366)
Supplement: Supplementary file 1 [file ao5c09366_si_001.pdf]

Gas Sensing Properties of Novel Indium Oxide monolayer: A First-Principles Study

Afreen Anamul Haque, Suraj G. Dhongade, and Aniket Singha*

Department of Electronics and Electrical Communication Engineering, Indian Institute of Technology Kharagpur, Kharagpur-721302, India

E-mail: aniket@ece.iitkgp.ac.in

A1. Methodology for Initial Adsorption Site Selection and Structural Optimization

The adsorption of gas molecules on a monolayer surface preferentially occurs at the site with the most negative adsorption energy, corresponding to the most thermodynamically stable configuration of the gas–monolayer system. To identify such a configuration, the system was subjected to structural relaxation by initially placing the gas molecule at various high-symmetry sites on the monolayer surface. These initial adsorption sites were systematically selected based on symmetry considerations, as illustrated in Fig. S1: (i) atop a top-layer Indium (In) atom, (ii) atop a top-layer Oxygen (O) atom, (iii) above a middle-layer Oxygen atom (hollow site), (iv) at the midpoint of the bond between a top-layer In and O atom, and (v) at the midpoint of the bridge between two top-layer In atoms. Gas molecules were placed in vertical, horizontal and oblique (at 45^0 angle) orientations at each of these five adsorption sites. For polar molecules such as ammonia (NH_3) and water (H_2O), the effect of molecular dipole orientation was explored by aligning different molecular ends towards the surface. An initial vertical separation of 2.5 Å was maintained between each molecule and the surface. Structural relaxation was subsequently performed for all initial configurations, and the most energetically favorable structure was determined to represent the most stable adsorption configuration for each gas species. Due to the symmetry of the hexagonal lattice, certain geometries converged to equivalent energy minima. This comprehensive methodology enabled the determination of the most favorable adsorption sites and configurations based on minimum energy criteria. Representative top and side views of the lowest-energy configurations for all ten harmful gas molecules and three ambient molecules under consideration are presented in Figure S1.

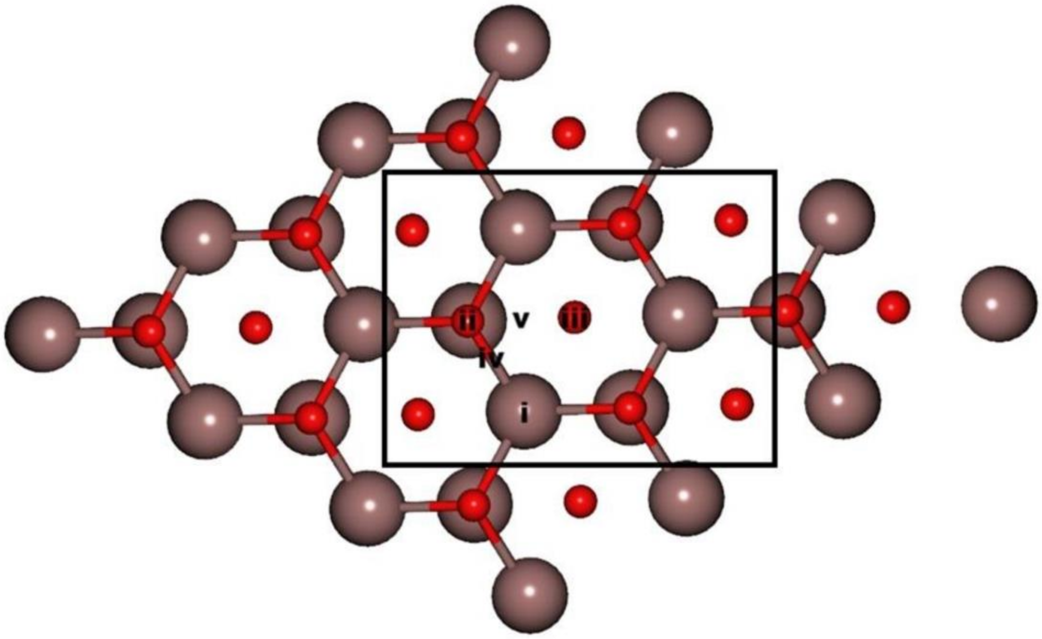

Fig. S1: Top view of a 3 × 3 × 1 In_2O_3 monolayer supercell, highlighting the identified initial adsorption sites (high symmetry sites): (i) atop a top-layer Indium (In) atom, (ii) atop a top-layer Oxygen (O) atom, (iii) above a middle-layer Oxygen atom (hollow site), (iv) at the midpoint of the bond between a top-layer In and O atom, and (v) at the midpoint of the bridge between two top-layer In atoms. Atom color coding: Indium (In) – greyish brown; Oxygen (O) – red.

A2. Top and side views of the monolayer with adsorbed molecules in the most stable configuration

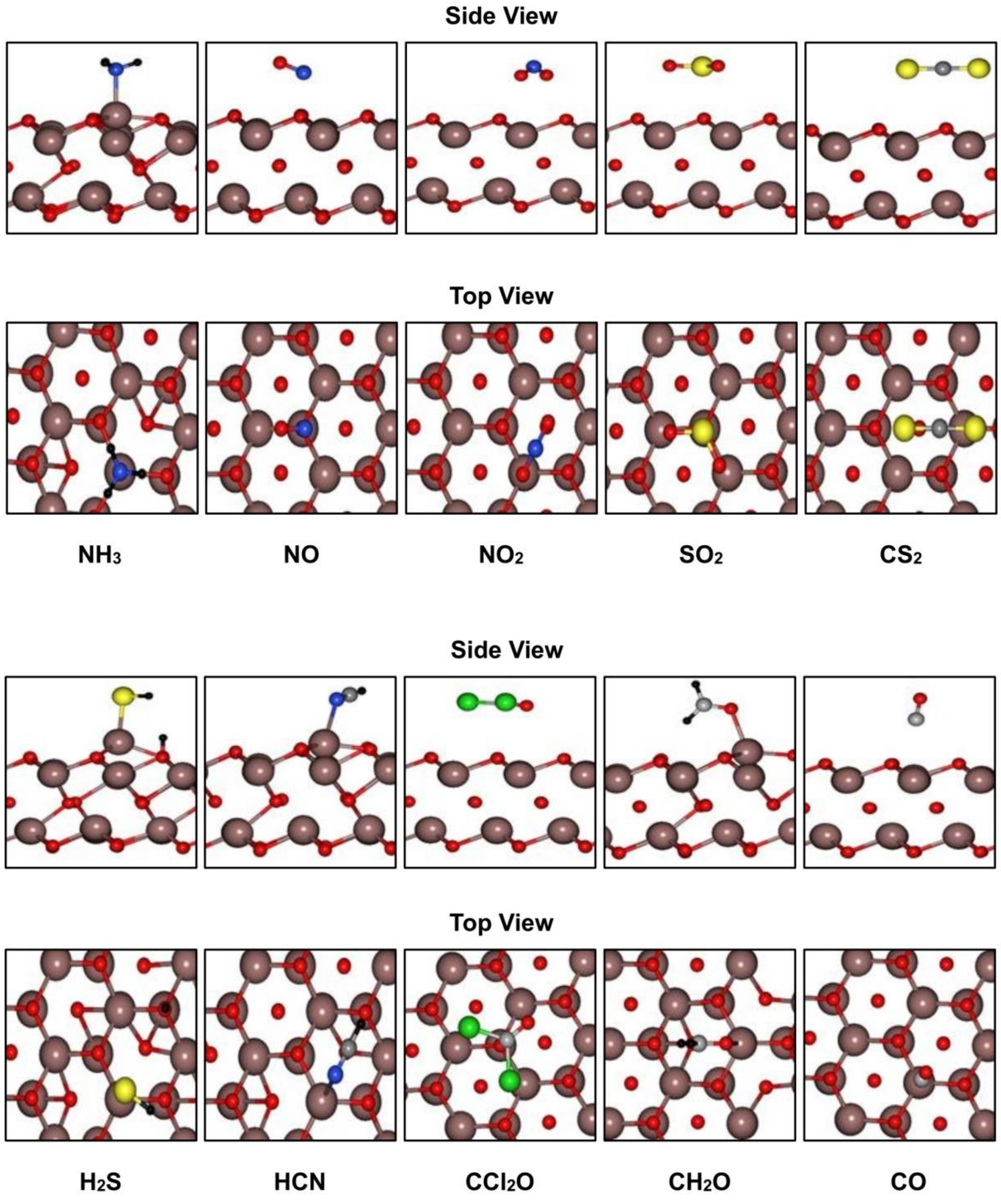

Fig. S2: Top and side views of the most stable adsorption configurations of the In_2O_3 monolayer for the ten toxic gas molecules under investigation. Atoms are color-coded as follows: Indium (In) – greyish brown, Oxygen (O) – red, Nitrogen (N) – blue, Hydrogen (H) – black, Carbon (C) – grey, Sulfur (S) – yellow, and Chlorine (Cl) – green.

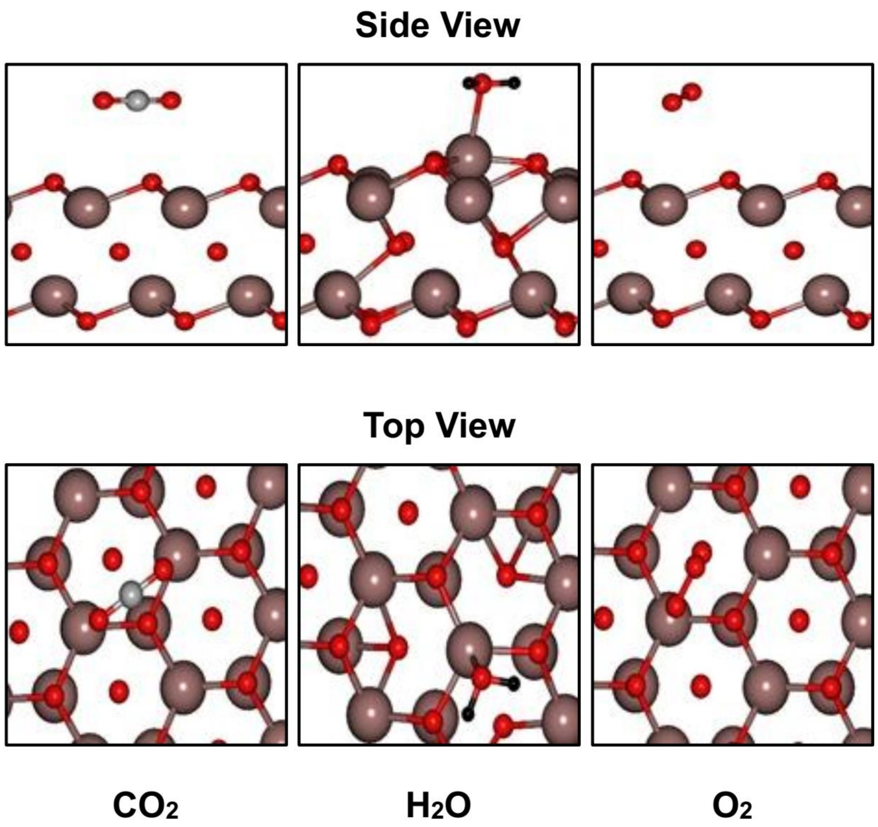

Fig. S3: Top and side views of the most stable adsorption configurations of the In_2O_3 monolayer for the three atmospheric gas molecules under study. Atoms are color-coded as follows: Indium (In) – greyish brown, Oxygen (O) – red, Hydrogen (H) – black, and Carbon (C) – grey.

A3: Comparison of gas adsorption energy and recovery time from literature with In_2O_3 monolayer for NH_3, NO, H_2S, HCN, CH_2O and H_2O molecules which demonstrate E_{ads}<-0.4eV

TABLE S1

THEORETICAL STUDY OF THE NH_3 ADSORPTION ON DIFFERENT 2D MATERIALS FROM THE LITERATURE, HIGHLIGHTING ADSORPTION ENERGY AND RECOVERY TIME BASED ON DFT CALCULATIONS.

System	E_{ads} (eV)	τ (s)	Functional
In_2Se_3 (bottom) [1]	-0.49	1.70 x 10^{-4}	GGA-PBE
In_2Se_3 (top) [1]	-0.19	1.56 x 10^{-9}	GGA-PBE
MoS_2 [2]	-0.02	2.53 x 10^{-12}	GGA-PBE
Cu-doped WS_2 [3]	-1.02	1.85 x 10^{5}	GGA-PBE
WS_2 [4]	0.31	5.96 x 10^{-18}	GGA-PBE
Graphene [5]	-0.11	7.05 x 10^{-11}	LDA
B-doped Graphene [5]	-0.50	2.51 x 10^{-4}	LDA
Phosphorene [6]	-0.18	1.06 x 10^{-9}	optB88
Silicene [7]	-0.60	1.20 x 10^{-2}	GGA-PBE
Hexagonal Ga_2O_3 (top) [8]	-1.19	1.06 x 10^{8}	GGA-PBE
Hexagonal Ga_2O_3 (bottom) [8]	-0.41	7.72 x 10^{-6}	GGA-PBE
Ga_2O_2 [9]	-0.54	1.45 x 10^{-3}	GGA-PBE
In_2O_3 (this work)	-1.07	8.17 x 10^{5}	GGA-PBE

TABLE S2

THEORETICAL STUDY OF THE NO ADSORPTION ON DIFFERENT 2D MATERIALS FROM THE LITERATURE, HIGHLIGHTING ADSORPTION ENERGY AND RECOVERY TIME BASED ON DFT CALCULATIONS.

System	E_{ads} (eV)	τ (ms)	Functional
In_2Se_3 (bottom) [1]	-0.19	1.63 × 10^{-6}	GGA-PBE
In_2Se_3 (top) [1]	-0.12	9.89 x 10^{-8}	GGA-PBE
MoS_2 [2]	-0.03	3.61 x 10^{-9}	GGA-PBE
WS_2 [4]	-0.25	1.58 x 10^{-7}	GGA-PBE
Graphene [5]	-0.30	1.18 x 10^{-4}	LDA
B-doped Graphene [5]	-1.07	1.25 x 10^{3}	LDA
Phosphorene [6]	-0.32	2.58 x 10^{-4}	optB88
Silicene [7]	-0.57	4.36	GGA-PBE
Hexagonal Ga_2O_3 (top) [8]	-0.60	19.2	GGA-PBE
Hexagonal Ga_2O_3 (bottom) [8]	-0.15	3.31 x 10^{-7}	GGA-PBE
Ga_2O_2	-0.26	2.76 x 10^{-5}	GGA-PBE
In_2O_3 (this work)	-0.68	197	GGA-PBE

TABLE S3

THEORETICAL STUDY OF THE H_2S ADSORPTION ON DIFFERENT 2D MATERIALS FROM THE LITERATURE, HIGHLIGHTING ADSORPTION ENERGY AND RECOVERY TIME BASED ON DFT CALCULATIONS.

System	E_{ads} (eV)	τ (s)	Functional
Penta $PdAs_2$ (bottom) [10]	-0.49	1.79 x 10^{-6}	GGA-PBE
MoWC based MXenes (Mo side) [11]	-1.15	2.213 x 10^{7}(300K)	GGA-PBE
MoWC based MXenes (W side) [11]	-0.95	1.311 x 10^{4}(300K)	GGA-PBE
MoWCO2 based MXenes (Mo side) [11]	-0.29	8.543 x 10^{-8}(300K)	GGA-PBE
MoWCO2 based MXenes (W side) [11]	-0.32	5.222 x 10^{-9}(300K)	GGA-PBE
GeC [12]	-0.20	3.04 x 10^{-9}	GGA-PBE
CoOOH [13]	-0.43	-	GGA-PBE
Hexagonal Ga_2O_3 (top) [14]	-0.19	-	GGA-PBE
Hexagonal Ga_2O_3 (bottom) [14]	-0.31	-	GGA-PBE
Graphene [15]	-0.61	-	GGA-PBE
Au-doped Graphene [15]	-0.90	-	GGA-PBE
Bilayer Graphene [16]	-0.16	-	GGA-PBE
Pristine $MoSe_2$ [17]	-0.35	7.59 x 10^{-7} (300K)	GGA-PBE
Ru-doped $MoSe_2$ [17]	-1.13	9.63 x 10^{6} (300K)	GGA-PBE
Rh-doped $MoSe_2$ [17]	-1.04	2.96 x 10^{5} (300K)	GGA-PBE
Pd-doped $MoSe_2$ [17]	-1.02	1.36 x 10^{5} (300K)	GGA-PBE
MoO_3 [18]	-0.5	-	GGA-PBE
In_2O_3 (this work)	-1.29	3.63 x 10^{9}	GGA-PBE

TABLE S4

THEORETICAL STUDY OF THE HCN ADSORPTION ON DIFFERENT 2D MATERIALS FROM THE LITERATURE, HIGHLIGHTING ADSORPTION ENERGY AND RECOVERY TIME BASED ON DFT CALCULATIONS.

System	E_{ads} (eV)	τ (s)	Functional
Pristine $MoSe_2$ [17]	-0.27	3.44 x 10^{-8}(300K)	GGA-PBE
Ru-doped $MoSe_2$ [17]	-1.71	6.39 (673K)	GGA-PBE
Rh-doped $MoSe_2$ [17]	-0.85	18.32(323K)	GGA-PBE
Pd-doped $MoSe_2$ [17]	-0.78	12.70 (300K)	GGA-PBE
Ta-doped $MoSe_2$ (W side) [19]	-0.98	4.64 (390K)	GGA-PBE
$B_6N_6H_6$ [20]	-0.31	1.00 x 10^{-6}(300K)	GGA-PBE
CoOOH [13]	-0.80	339 (298K)	GGA-PBE
Al-doped Graphene [21]	-1.67	-	X3LYP
Si-doped Graphene [21]	-1.17	-	X3LYP
Phosphorene [22]	-0.24	-	GGA-PBE
Al-doped Phosphorene [22]	-0.59	-	GGA-PBE
Si-doped Phosphorene [22]	-0.19	-	GGA-PBE
S-doped Phosphorene [22]	-0.19	-	GGA-PBE
In_2O_3 (this work)	-0.46	5.43 x 10^{-5}	GGA-PBE

TABLE S5

THEORETICAL STUDY OF THE CH_2O ADSORPTION ON DIFFERENT 2D MATERIALS FROM THE LITERATURE, HIGHLIGHTING ADSORPTION ENERGY AND RECOVERY TIME BASED ON DFT CALCULATIONS.

System	E_{ads} (eV)	τ (s)	Functional
BN [23]	-0.28	5.06 x 10^{-8}	GGA-PBE
AlN [23]	-1.04	2.96 x 10^{5}	GGA-PBE
GaN [23]	-0.46	5.34 x 10^{-5}	GGA-PBE
InN [23]	-1.05	4.36 x 10^{5}	GGA-PBE
BP [23]	-0.25	1.58 x 10^{-8}	GGA-PBE
P [23]	-0.19	1.56 x 10^{-9}	GGA-PBE
GeC [24]	-0.27	8.76 x 10^{-10}	GGA-PBE
2D B_3O_3 [25]	-0.35	8.1 × 10^{-7}	GGA-PBE
2D Ga_2O_2 [9]	-0.64	5.64 x 10^{-2}	GGA-PBE
2D In_2O_3 (this work)	-0.64	5.64 x 10^{-2}	GGA-PBE

TABLE S6

THEORETICAL STUDY OF THE H_2O ADSORPTION ON DIFFERENT 2D MATERIALS FROM THE LITERATURE, HIGHLIGHTING ADSORPTION ENERGY AND RECOVERY TIME BASED ON DFT CALCULATIONS.

System	E_{ads} (eV)	τ (s)	Functional
Monolayer MoS_2 edges [26]	-0.55	1.74 x 10^{-3}	GGA-PBE
2D Ferromagnetic Fe_3GeTe_2 [27]	-0.25	1.85 x 10^{-8}	GGA-PBE
defective-Fe_3GeTe_2 [27]	-0.76	5.85	GGA-PBE
Monolayer Pt(111) [28]	-0.42	1.14 x 10^{-5}	vdW-DF2-B86R
GeC [24]	-0.27	8.76 x 10^{-10}	GGA-PBE
2D Ga_2O_2 [9]	-0.19	1.94 × 10^{-9}	GGA-PBE
2D In_2O_3 (this work)	-0.63	3.83 x 10^{-2}	GGA-PBE

<u>A4. DOS Profiles and conductivity change factor of Gas-Adsorbed In_2O_3 Monolayer Systems as computed with hybrid HSE computation.</u>

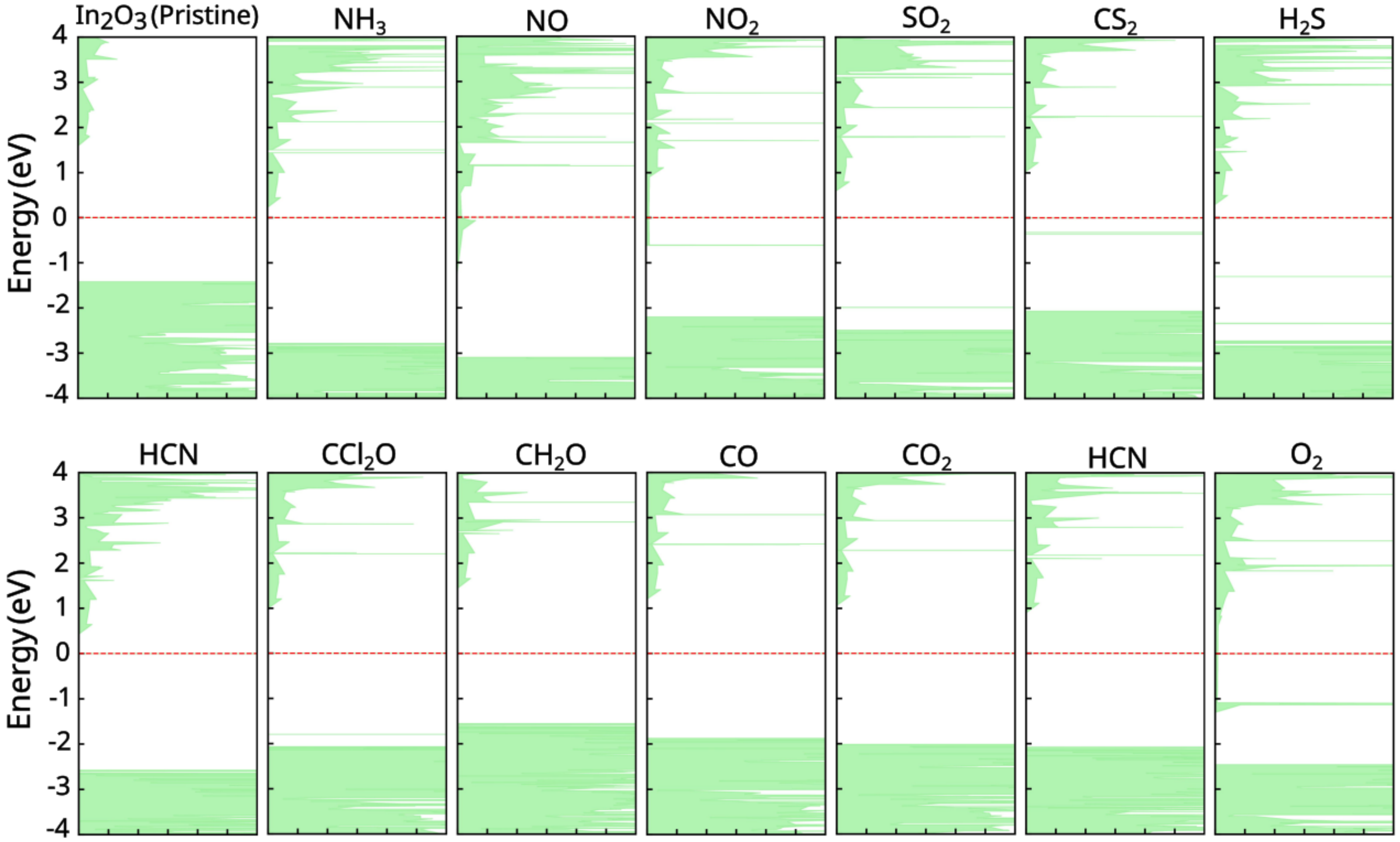

Fig. S4: Total density of states (TDOS) profiles for the molecule–monolayer systems under investigation, as computed using hybrid HSE calculations. In all cases, the intrinsic Fermi level is indicated by a red dashed line.

The DOS profile of pristine and gas adsorbed In_2O_3 monolayer is depicted in Fig. S4. We note that in all the cases, the DOS profile or trend remain unchanged, except for a change in band-gap. We also note additional induced state in the DOS profile of CS_2 adsorbed monolayer (about 1.3eV below the conduction band edge), in addition to spreading out of the induced states in NO_2 and O_2 adsorbed monolayer over the energy axis. However, this doesn't alter the overall trend or conclusion presented in the main manuscript. NO_2, CS_2 and O_2 demonstrate adsorption energy of -0.29eV, -0.33eV and -0.13eV, which are not strong enough to induce stable measurable signal at room temperature. The values of χ as computed from the DOS obtained using hybrid HSE calculations are given in Table S7 in the next page.

Table S7

Comparison of E_g and χ obtained from GGA-PBE and hybrid-HSE calculations. We note that the trends obtained in E_g and χ are effectively the same for GGA-PBE and HSE computations, except for CS_2 adsorbed monolayer. The drastic difference in χ for CS_2 adsorption arises because HSE (unlike GGA–PBE) induces a filled electronic state close to the conduction-band edge.

**Molecule**	**E_g (eV)**		**χ**	
	**PBE**	**HSE**	**PBE**	**HSE**
Pristine In_2O_3	1.65	2.98	1.0	1.0
NH_3	1.59	2.98	3.19	1.0
NO	0	0	Very high	Very high
NO_2	0.3	0	2.13×10^{11}	Very high
SO_2	1.24	2.55	2.76×10^{3}	4.6×10^{3}
CS_2	1.66	1.3	0.82	1.25×10^{14}
H_2S	0.59	1.55	7.86×10^{8}	1.00×10^{12}
HCN	1.60	2.97	2.63	1.21
CCl_2O	1.66	2.79	0.82	39.31
CH_2O	1.57	2.94	4.69	2.16
CO	1.65	2.97	1.0	1.21
CO_2	1.65	2.98	1.0	1.0
H_2O	1.55	2.95	6.91	1.78
O_2	0	0	Very high	Very high

A5. DOS Profiles of Gas-Adsorbed In_2O_3 Monolayer Systems Under 3% Tensile Strain

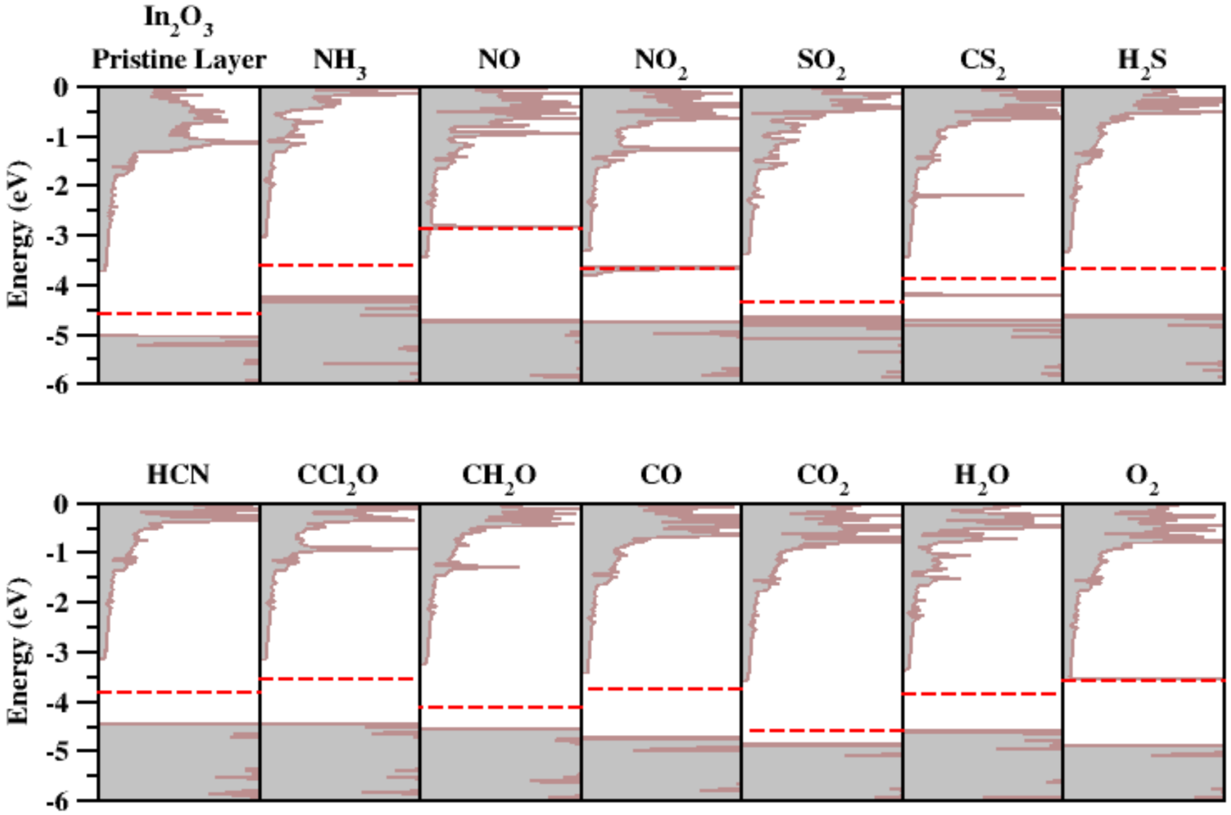

Fig. S5: Total density of states (TDOS) profiles for the molecule–monolayer systems under investigation, with 3% tensile strain applied to the In_2O_3 monolayer. In all cases, the intrinsic Fermi level is indicated by a red dashed line.

A6. DOS Profiles of Gas-Adsorbed In_2O_3 Monolayer Systems Under 2% Compressive Strain

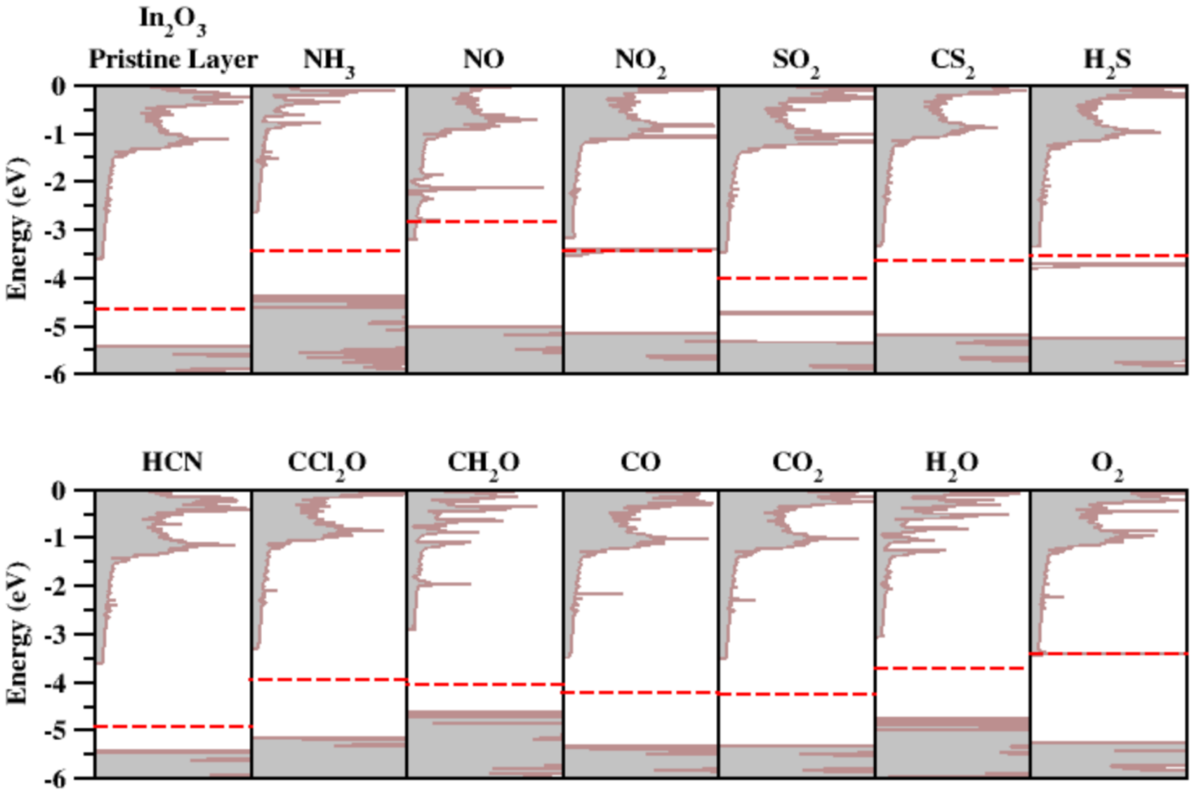

Fig. S6: Total density of states (TDOS) profiles for the molecule–monolayer systems under investigation, with 2% compressive strain applied to the In_2O_3 monolayer. In all cases, the intrinsic Fermi level is indicated by a red dashed line.

A7. Average Potential Profiles of 2D In_2O_3 Monolayer–Gas Molecule Configurations Without Applied Strain

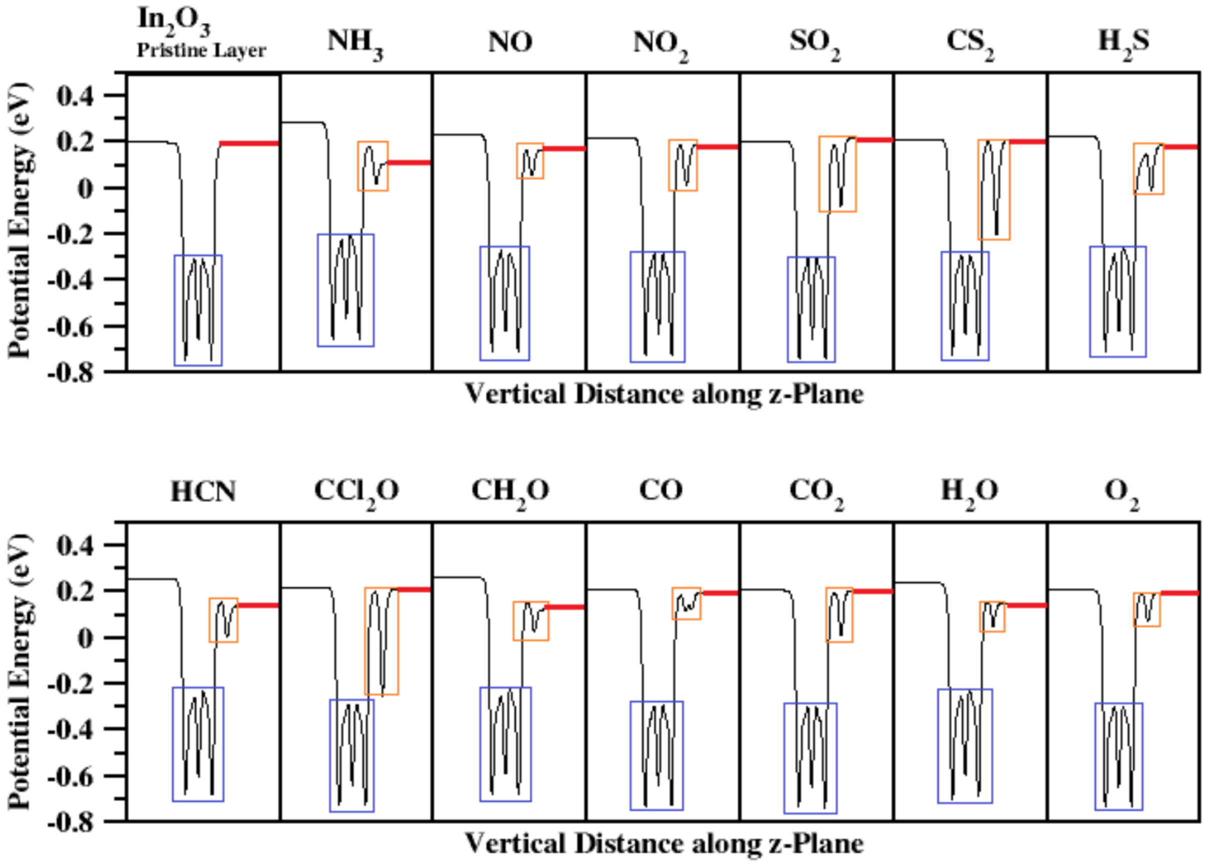

Fig. S7: Average electrostatic potential profile for the pristine In_2O_3 monolayer and the gas-adsorbed configurations of the monolayer without applied strain. The blue-shaded region corresponds to the potential variation due to the atoms of the 2D monolayer, while the amber-shaded region represents the effect of the adsorbed gas molecule on the potential profile. The potential is plotted along the axis perpendicular to the monolayer plane (x-axis). The red horizontal line denotes the vacuum potential of the surface with the adsorbed gas molecule, and is used as E_{vac} in Eqn (6) for the calculation of the work function in the main paper. $\phi = E_{vac} - E_f$

A8. Average Potential Profiles of 2D In_2O_3 Monolayer–Gas Molecule Configurations Under 3% Tensile Strain

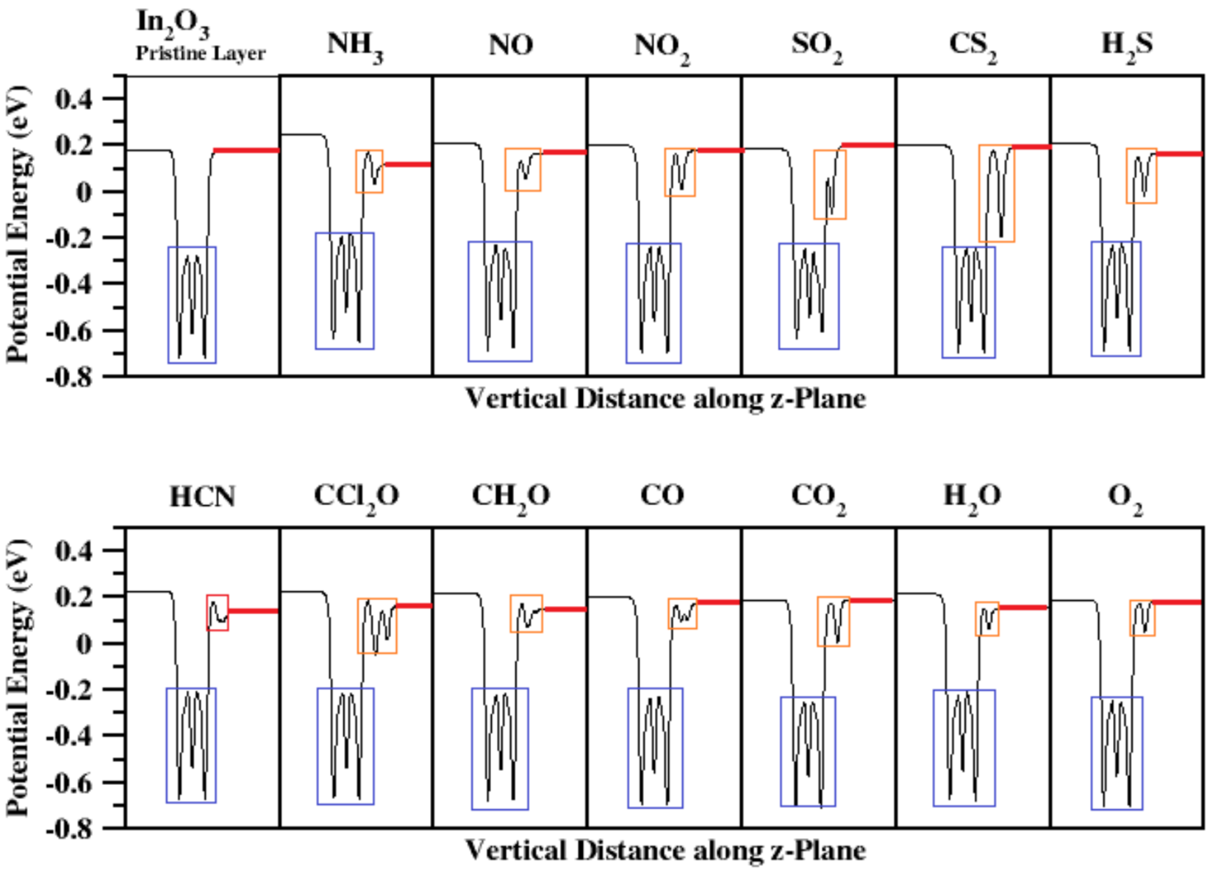

Fig. S8: Average electrostatic potential profile for the pristine In_2O_3 monolayer and the gas-adsorbed configurations of the monolayer with 3% tensile strain. The blue-shaded region corresponds to the potential variation due to the atoms of the 2D monolayer, while the amber-shaded region represents the effect of the adsorbed gas molecule on the potential profile. The potential is plotted along the axis perpendicular to the monolayer plane (x-axis). The red horizontal line denotes the vacuum potential of the surface with the adsorbed gas molecule, and is used as E_{vac} in Eqn (6) for the calculation of the work function in the main paper: $\phi = E_{vac} - E_f$

A9. Average Potential Profiles of 2D In_2O_3 Monolayer–Gas Molecule Configurations Under 2% Compressive Strain

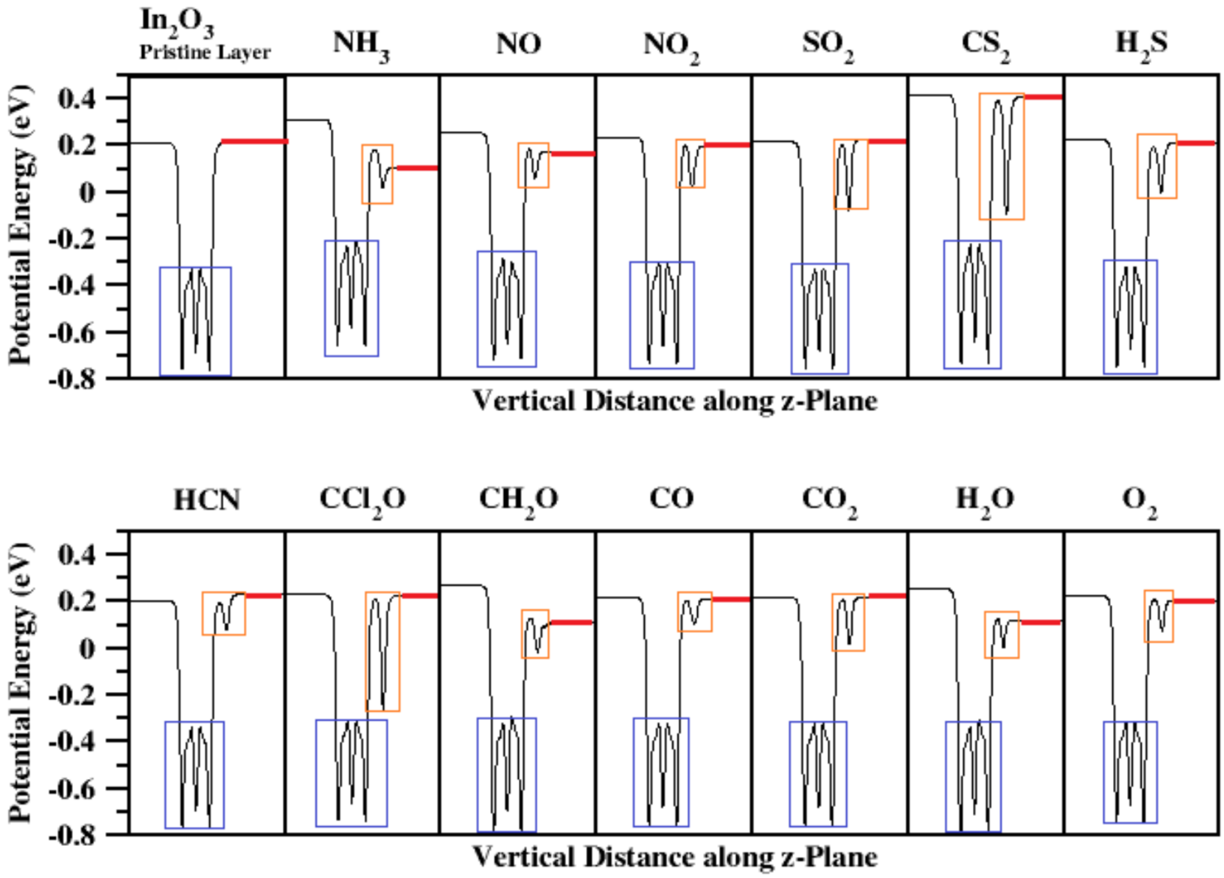

Fig. S9: Average electrostatic potential profile for the pristine In_2O_3 monolayer and the gas-adsorbed configurations of the monolayer with 2% compressive strain. The blue-shaded region corresponds to the potential variation due to the atoms of the 2D monolayer, while the amber-shaded region represents the effect of the adsorbed gas molecule on the potential profile. The potential is plotted along the axis perpendicular to the monolayer plane (x-axis). The red horizontal line denotes the vacuum potential of the surface with the adsorbed gas molecule, and is used as E_{vac} in Eqn (6) for the calculation of the work function in the main paper: $\phi = E_{vac} - E_f$

References

[1] Q. Yue, Z. Shao, S. Chang, and J. Li, “Adsorption of gas molecules on monolayer mos2 and effect of applied electric field,” *Nanoscale research letters*, vol. 8, no. 1, pp. 1–7, 2013.

[2] D. Zhao, Y. Wen, Z. Li, Y. Cui, Y. Zhao, T.-F. Lu, M. He, B. Song, and Z. Zhang, “Theoretical study of adsorption of gas (co, co2, nh3) by metal (au, ag, cu)-doped single-layer ws2,” *Journal of Molecular Modeling*, vol. 30, no. 10, p. 322, 2024.

[3] V. Q. Bui, T.-T. Pham, D. A. Le, C. M. Thi, and H. M. Le, “A first-principles investigation of various gas (co, h2o, no, and o2) absorptions on a ws2 monolayer: stability and electronic properties,” *Journal of Physics: Condensed Matter*, vol. 27, no. 30, p. 305005, 2015.

[4] Z. Yonghui, C. Yabin, Z. Kaige, L. Caihong, Z. Jing, Z. Haoli, E. Peng Yong *et al.*, “Improving gas sensing properties of graphene by introducing dopants and defects: a first-principles study,” *Nanotechnology (Print)*, vol. 20, 2009.

[5] Y. Cai, Q. Ke, G. Zhang, and Y.-W. Zhang, “Energetics, charge transfer, and magnetism of small molecules physisorbed on phosphorene,” *The Journal of Physical Chemistry C*, vol. 119, no. 6, pp. 3102–3110, 2015.

[6] W. Hu, N. Xia, X. Wu, Z. Li, and J. Yang, “Silicene as a highly sensitive molecule sensor for nh 3, no and no 2,” *Physical Chemistry Chemical Physics*, vol. 16, no. 15, pp. 6957–6962, 2014.

[7] J. Zhao, X. Huang, Y. Yin, Y. Liao, H. Mo, Q. Qian, Y. Guo, X. Chen, Z. Zhang, and M. Hua, “Two-dimensional gallium oxide monolayer for gas-sensing application,” *The Journal of Physical Chemistry Letters*, vol. 12, no. 24, pp. 5813–5820, 2021.

[8] P. Bergveld, J. Hendrikse, and W. Olthuis, “Theory and application of the material work function for chemical sensors based on the field effect principle,” *Measurement science and technology*, vol. 9, no. 11, p. 1801, 1998.

[9] A. A. Haque, S. G. Dhongade, and A. Singha, “Predictive analysis of gas sensing properties in a novel 2d gallium oxide phase,” *IEEE Sensors Journal*, vol. 25, no. 8, pp. 12 644–12 652, 2025.

[10] D. Raval, S. K. Gupta, and P. Gajjar, “Detection of h2s, hf and h2 pollutant gases on the surface of penta-pdas2 monolayer using dft approach,” *Scientific Reports*, vol. 13, no. 1, p. 699, 2023.

[11] S. Khammuang, A. Udomkijmongkol, S. Thasitha, T. Hussain, and K. Kotmool, “First-principles study insights into janus mowc-based mxenes for enhanced h2s and nh3 sensing applications,” *Applied Surface Science*, vol. 699, p. 163110, 2025. [Online]. Available: https://www.sciencedirect.com/science/article/pii/S0169433225008244

[12] S. Luo, R. Tu, W. Liu, J. Li, J. Li, K. Wang, J. Guo, S. Li, and X. Dai, “Adsorption of gas molecules (c2h6, co, h2s, ch2o, ch4, and co2) on gec monolayer: A first-principles study,” *Computational and Theoretical Chemistry*, vol. 1249, p. 115263, 2025. [Online]. Available: https://www.sciencedirect.com/science/article/pii/S2210271X25001999

[13] F. Opoku and P. P. Govender, “Two-dimensional coooh as a highly sensitive and selective h2s, hcn and hf gas sensor: A computational investigation,” *Electroanalysis*, vol. 32, no. 12, pp. 2764–2774, 2020. [Online]. Available: https://analyticalsciencejournals.onlinelibrary.wiley.com/doi/abs/10.1002/elan.202060337

[14] J. Zhu, Z. Xu, S. Ha, D. Li, K. Zhang, H. Zhang, and J. Feng, “Gallium oxide for gas sensor applications: A comprehensive review,” *Materials*, vol. 15, no. 20, 2022. [Online]. Available: https://www.mdpi.com/1996-1944/15/20/7339

[15] X. Zhang, L. Yu, X. Wu, and W. Hu, “Experimental sensing and density functional theory study of h2s and sof2 adsorption on au-modified graphene,” *Advanced Science*, vol. 2, no. 11, p. 1500101, 2015. [Online]. Available: https://advanced.onlinelibrary.wiley.com/doi/abs/10.1002/advs.201500101

[16] R. Majidi and A. Ramazani, “Detection of hf and h2s with pristine and ti-embedded twin graphene: A density functional theory study,” *Journal of Physics and Chemistry of Solids*, vol. 132, pp. 31–37, 2019. [Online]. Available: https://www.sciencedirect.com/science/article/pii/S0022369719302781

[17] N. Viveka, C. Poornimadevi, C. P. Kala, and D. J. Thiruvadigal, “Dft insights into the gas sensing properties of light platinum group metal (ru, rh & pd) doped mose2 monolayers,” *Surfaces and Interfaces*, vol. 66, p. 106579, 2025. [Online]. Available: https://www.sciencedirect.com/science/article/pii/S2468023025008363

[18] K. Xu, N. Liao, B. Zheng, and H. Zhou, “Adsorption and diffusion behaviors of h2, h2s, nh3, co and h2o gases molecules on moo3 monolayer: A dft study,” *Physics Letters A*, vol. 384, no. 21, p. 126533, 2020. [Online]. Available: https://www.sciencedirect.com/science/article/pii/S037596012030400X

[19] B. Liang, W. Li, Q. Ren, C. Zhu, and J. Li, “Gas adsorption performance of ta doped mose2 based on first principles,” *Results in Physics*, vol. 42, p. 105978, 2022. [Online]. Available: https://www.sciencedirect.com/science/article/pii/S2211379722005927

[20] Y. Yong, R. Gao, X. Wang, X. Yuan, S. Hu, Z. Zhao, X. Li, and Y. Kuang, “Highly sensitive and selective room-temperature gas sensors based on b6n6h6 monolayer for sensing so2 and nh3: A first-principles study,” *Results in Physics*, vol. 33, p. 105208, 2022. [Online]. Available: https://www.sciencedirect.com/science/article/pii/S2211379722000249

[21] S. F. Rastegar, A. A. Peyghan, and N. L. Hadipour, “Response of si- and al-doped graphenes toward hcn: A computational study,” *Applied Surface Science*, vol. 265, pp. 412–417, 2013. [Online]. Available: https://www.sciencedirect.com/science/article/pii/S0169433212019794

[22] J. Pang, Q. Yang, X. Ma, L. Wang, C. Tan, D. Xiong, H. Ye, and X. Chen, “Dft coupled with negf study of ultra-sensitive hcn and hnc gases detection and distinct i–v response based on phosphorene,” *Phys. Chem. Chem. Phys.*, vol. 19, pp. 30 852–30 860, 2017. [Online]. Available: http://dx.doi.org/10.1039/C7CP03941G

[23] C. Feng, H. Qin, D. Yang, and G. Zhang, “First-principles investigation of the adsorption behaviors of ch2o on bn, aln, gan, inn, bp, and p monolayers,” *Materials*, vol. 12, no. 4, 2019. [Online]. Available: https://www.mdpi.com/1996-1944/12/4/676

[24] S. Luo, R. Tu, W. Liu, J. Li, J. Li, K. Wang, J. Guo, S. Li, and X. Dai, “Adsorption of gas molecules (c2h6, co, h2s, ch2o, ch4, and co2) on gec monolayer: A first-principles study,” *Computational and Theoretical Chemistry*, vol. 1249, p. 115263, 2025. [Online]. Available: https://www.sciencedirect.com/science/article/pii/S2210271X25001999

[25] N. U. Rahman, A. A. Khan, R. Ullah, R. Ahmad, and I. Ahmad, “Selective sensing of nh3 and ch2o molecules by novel 2d porous hexagonal boron oxide (b3o3) monolayer: A dft approach,” *Surfaces and Interfaces*, vol. 29, p. 101767, 2022. [Online]. Available: https://www.sciencedirect.com/science/article/pii/S2468023022000487

[26] K. K. Ghuman, S. Yadav, and C. V. Singh, “Adsorption and dissociation of h2o on monolayered mos2 edges: Energetics and mechanism from ab initio simulations,” *The Journal of Physical Chemistry C*, vol. 119, no. 12, pp. 6518–6529, 2015. [Online]. Available: https://doi.org/10.1021/jp510899m

[27] Z. Cui, C. Xiao, Y. Lv, Q. Li, R. Sa, and Z. Ma, “Adsorption behavior of co, co2, h2, h2o, no, and o2 on pristine and defective 2d monolayer ferromagnetic fe3gete2,” *Applied Surface Science*, vol. 527, p. 146894, 2020. [Online]. Available: https://www.sciencedirect.com/science/article/pii/S0169433220316512

[28] J. Haruyama, T. Sugimoto, and O. Sugino, “First-principles study of water adsorption monolayer on pt(111): Adsorption energy and second-order nonlinear susceptibility,” *Phys. Rev. Mater.*, vol. 7, p. 115803, Nov 2023. [Online]. Available: https://link.aps.org/doi/10.1103/PhysRevMaterials.7.115803
